# Biologically Active Diterpenoids in the *Clerodendrum* Genus—A Review

**DOI:** 10.3390/ijms231911001

**Published:** 2022-09-20

**Authors:** Łukasz Kuźma, Jan Gomulski

**Affiliations:** Department of Biology and Pharmaceutical Botany, Medical University of Lodz, Muszyńskiego 1, 90-151 Lodz, Poland

**Keywords:** antibacterial, anti-parasitic, *Clerodendrum* spp., cytotoxic, diterpenoids, insecticidal

## Abstract

One of the key areas of interest in pharmacognosy is that of the diterpenoids; many studies have been performed to identify new sources, their optimal isolation and biological properties. An important source of abietane-, pimarane-, clerodane-type diterpenoids and their derivatives are the members of the genus *Clerodendrum*, of the *Lamiaceae*. Due to their diverse chemical nature, and the type of plant material, a range of extraction techniques are needed with various temperatures, solvent types and extraction times, as well as the use of an ultrasound bath. The diterpenoids isolated from *Clerodendrum* demonstrate a range of cytotoxic, anti-proliferative, antibacterial, anti-parasitic and anti-inflammatory activities. This review describes the various biological activities of the diterpenoids isolated so far from species of *Clerodendrum* with the indication of the most active ones, as well as those from other plant sources, taking into account their structure in terms of their activity, and summarises the methods for their extraction.

## 1. Introduction

The name *Clerodendrum* is derived from two Greek words: *kleros* (destiny or chance) and *dendron* (tree) [1]. It probably has a dual meaning: in ancient times, some plants were believed to have healing properties, while others were poisonous [1].

The genus *Clerodendrum* was first described by Linneus in 1753, and this was followed by the species *C. infortunatum* [2]. This unusual genus was originally classified into the *Verbenaceae* family by Liang [3] and Munir [4], among others, but is now included in the *Lamiaceae* family [2]. It currently includes about 500 species [5] growing in warm temperate and tropical regions of Africa, southern and eastern Asia, as well as America and northern Australia [6]. The genus includes a range of deciduous or evergreen shrubs, small trees, perennial herbs and woody vines [7]; in addition, some species are subshrubs or herbs. Young branchlets are usually four-angled with simple leaves, which are opposite or, rarely, whorled. Inflorescences are loosely cymose or capitate, arranged in terminal or, rarely, axillary paniculate thyrses. The calyx is campanulate or cup shaped. The corolla has a slender tube, five spreading lobes and four stamens. The style has two acute stigmatic lobes. A fruiting calyx is partly inflated. The fruit is a type of drupe and has four one-seeded pyrenes [3].

The members of the *Clerodendrum* genus are grown as ornamental plants [1], and some demonstrate pesticidal or insecticidal properties [7]. Furthermore, many others have been recommended for use in treating pyrexia, asthma, common cold, hypertension, furunculosis, dysentery, rheumatism, mammitis, anorexia, leucoderma, leprosy, arthrophlogosis and toothache [8,9,10,11]. Due to these numerous therapeutic properties, many *Clerodendrum* species have been the subject of phytochemical investigations over the past 40 years, resulting in the extraction, isolation, purification and identification of a range of phytocompounds, including various mono- and sesquiterpene diterpenoids, triterpenoids, flavonoids and their glycosylated forms; in addition, various phenylethanoid glycosides, steroids and their glycoside derivatives, cyclohexylethanoids, anthraquinones and cyanogenic glycosides have been noted [12,13,14,15,16,17,18,19,20,21].

Diterpenes are natural plant-derived secondary metabolites with the general formula C_20_H_32_. They constitute a chemically diverse group of secondary metabolites which are biosynthesised in the flowering shoots, roots or rhizomes. Such plant diterpenoids most commonly occur in a cyclic form [22]. The diterpenoids are classified into abietane, caurane, caurene, clerodane and labdane types according to their main skeleton [23].

As a result of this structural variation, they also demonstrate a range of pharmacological and biological activities, including antitumor (paclitaxel) [24], positive inotropic (forskolin) [25], vasodilatory, and hypotensive (manool) properties [26], as well as antiplatelet potential (forskolin) [27]. Some diterpenoids also demonstrate antibacterial properties. For example, salvipisone and aethiopinone, isolated from *Salvia sclarea* roots, exhibit bactericidal activity against Gram-positive bacteria [28]. Taxodione and its unique derivative 7-2′-oxohexyl-taxodione, both isolated from *Salvia austriaca* roots, exhibit strong cytotoxic activities against various tumour cell lines [29]. Furthermore, taxodione inhibits acethylcholine- and butyrylcholine-esterase (ACE and BCE) [30].

The diterpenes are believed to exert cytotoxic activities through a range of possible mechanisms. Fronza et al. (2012) suggest that they exert their cytotoxic activity by targetting the biological membrane, with its lipophilic character [31]. Other abietane diterpenes could exert their cytotoxicity effects by their alkylating [31] and protonophoric properties [32]. In addition, sphaeropsidin A, a pimarane diterpene isolated from a fungal pathogen, was found to significantly affect cellular homeostasis by modulating the ion-transporter activity of the Na-K-2Cl electrochemical cotransporter or the Cl^−^/HCO^3−^ anion exchanger, thus increasing cellular volume [33].

**Figure 1:**
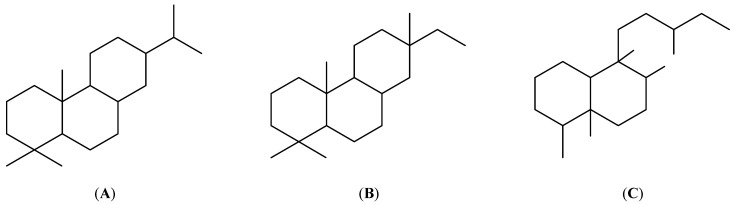
The structure of abietane (**A**), pimarane (**B**) and clerodane (**C**) diterpenoids.

Due to their diverse and often strong biological activities, diterpenoids make an interesting class of natural compounds. There are also attempts to identify new plant sources. A range of reports indicate that the roots and aerial parts of the *Clerodendrum* genus are rich in abietane-, pimarane- and clerodane-type diterpenoids and their derivatives, including their glycoside forms (Figure 1).

**Figure 2:**
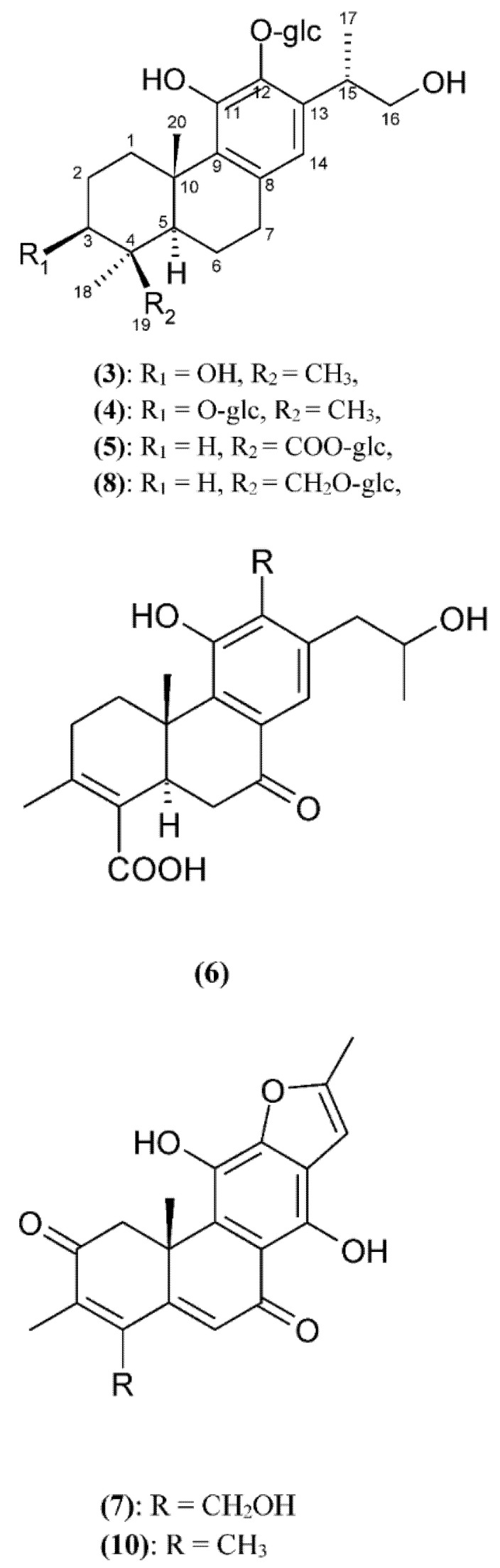

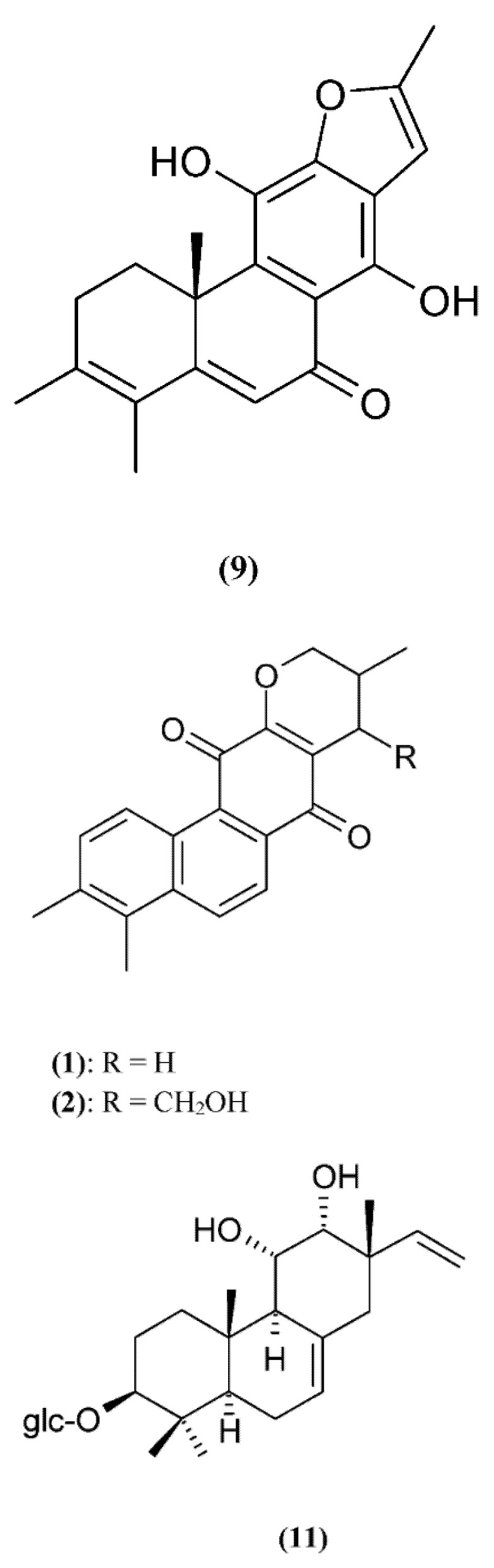

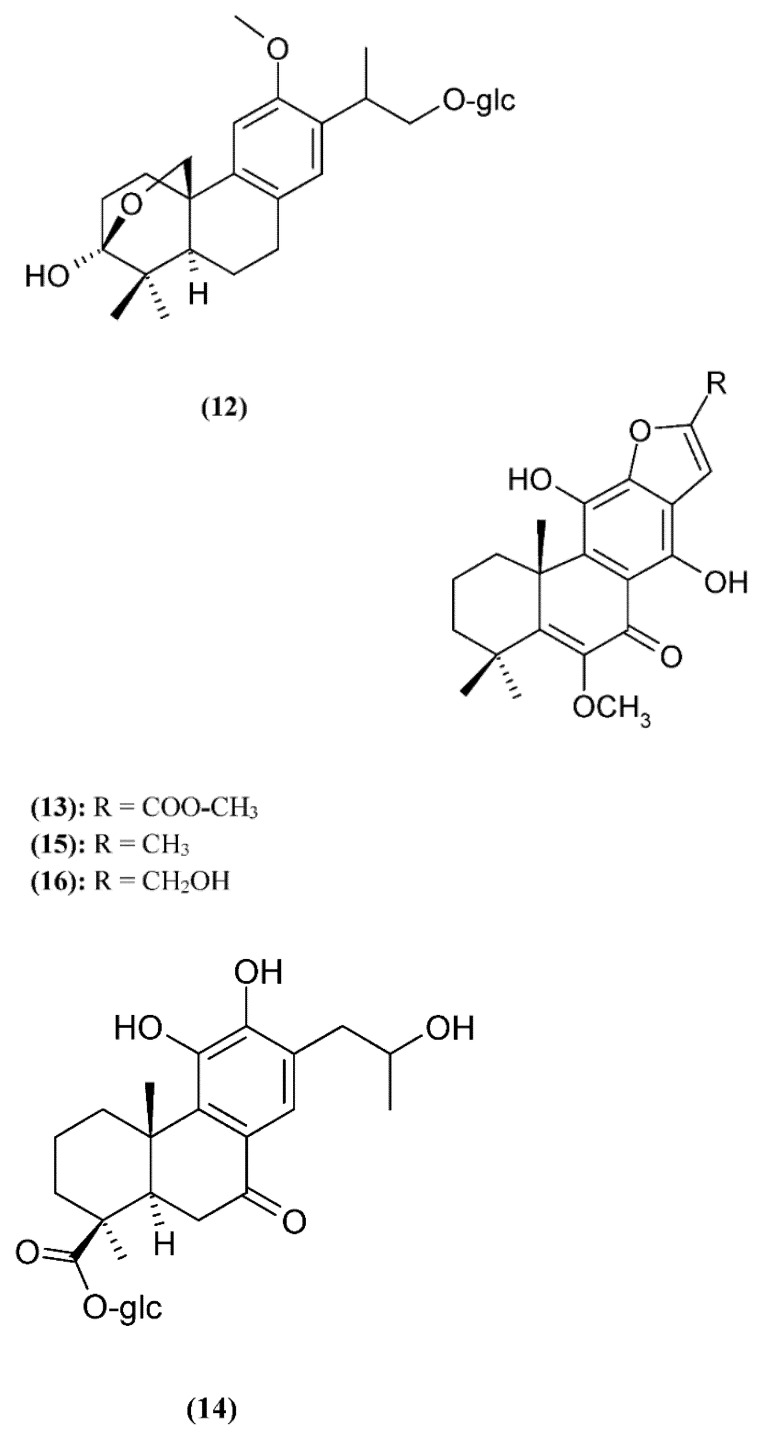

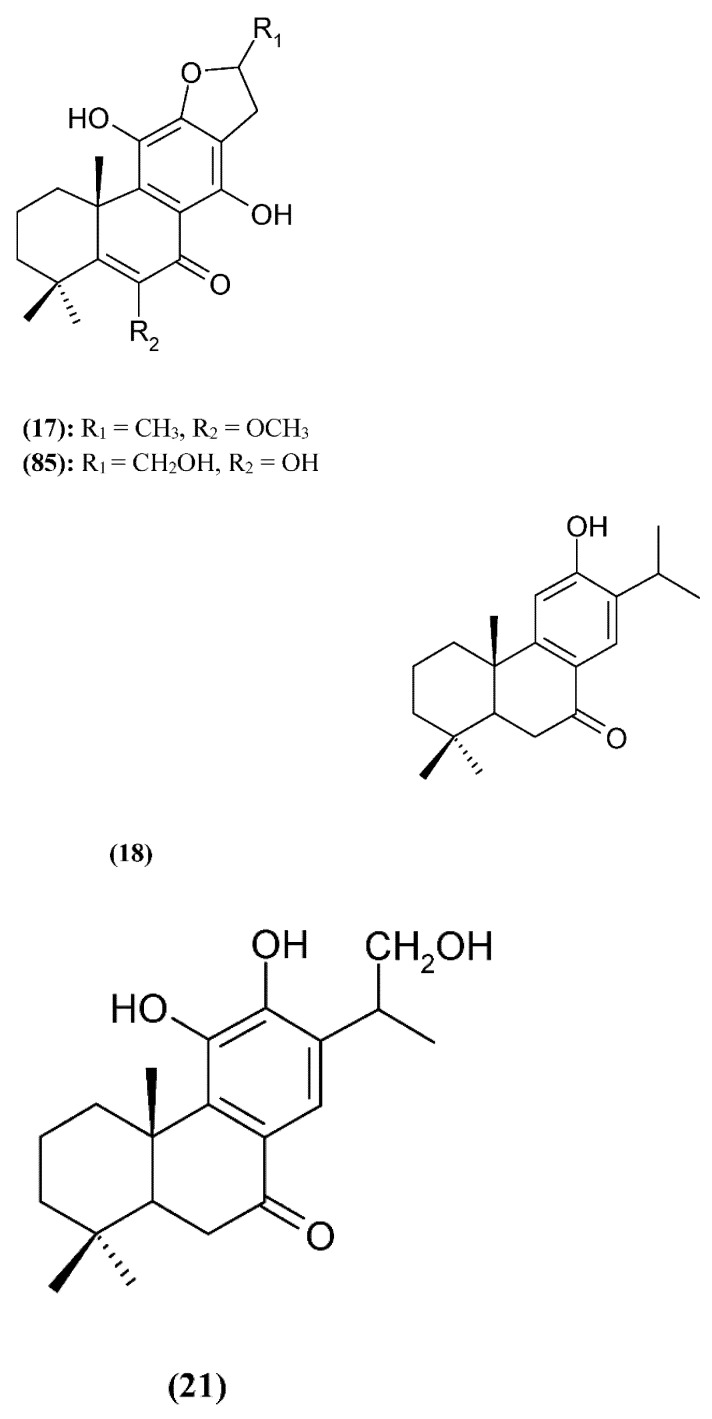

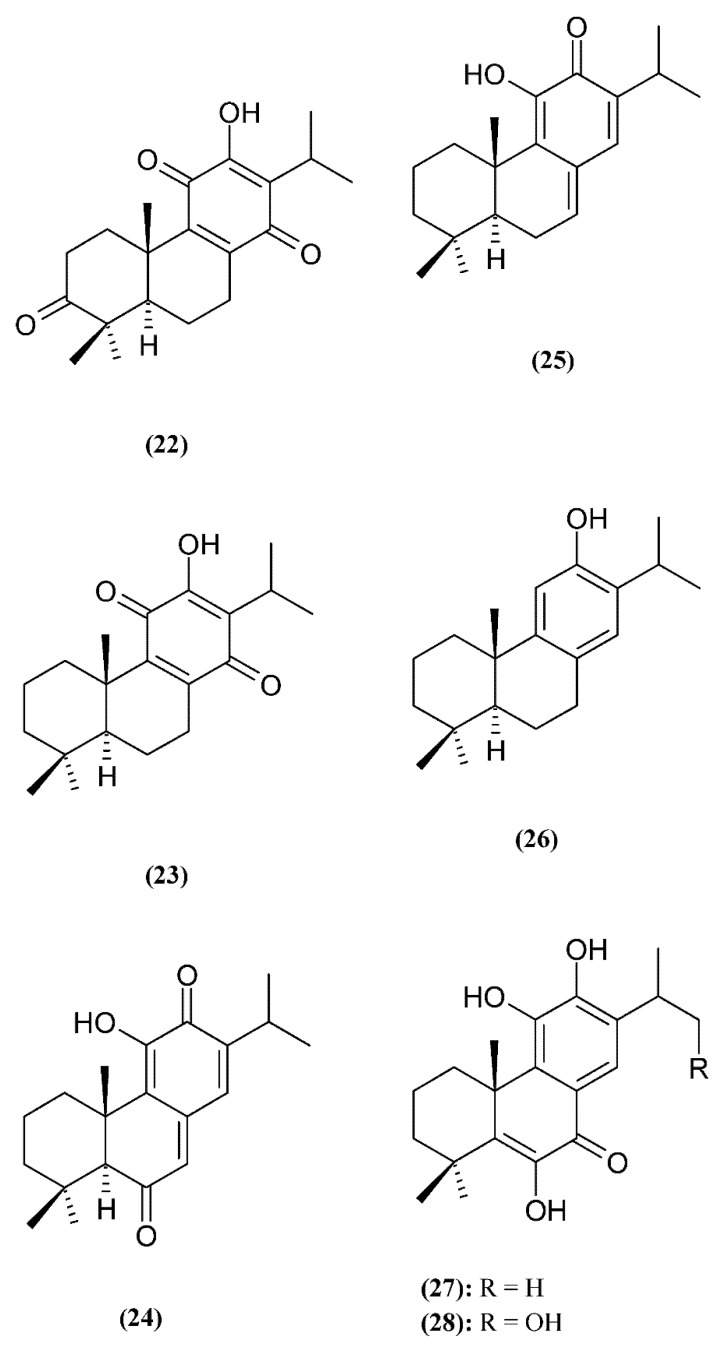

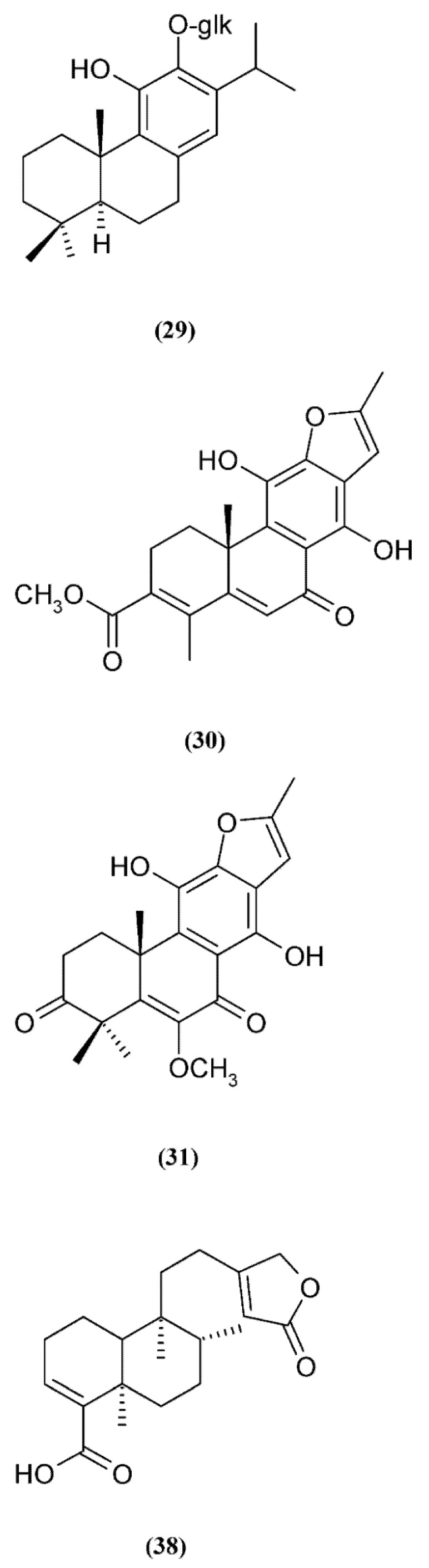

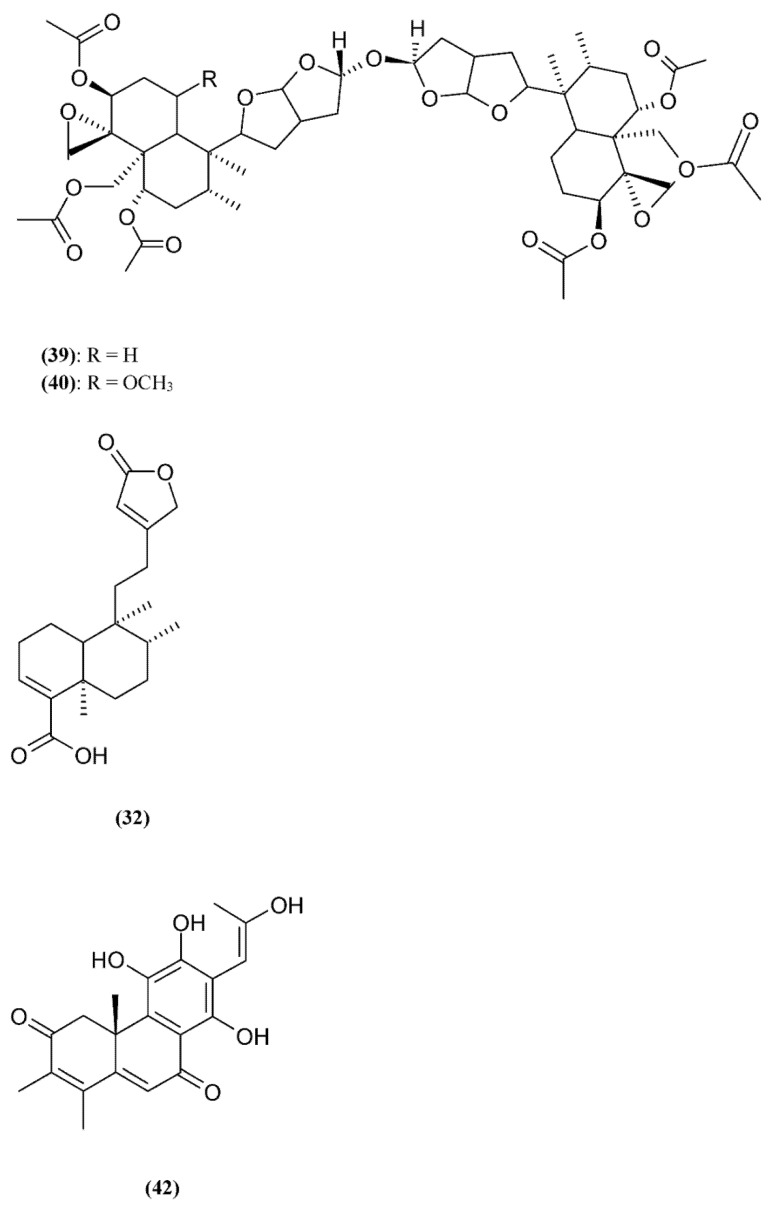

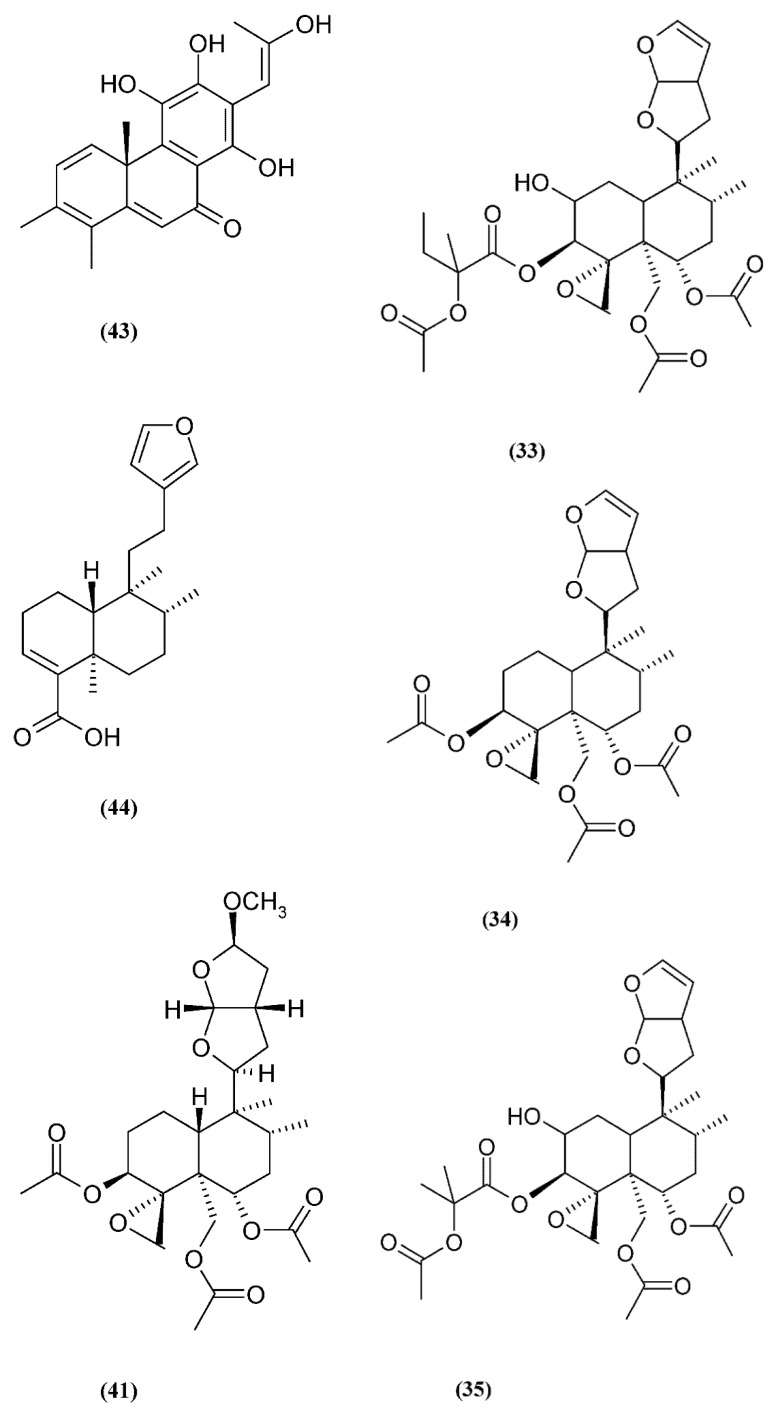

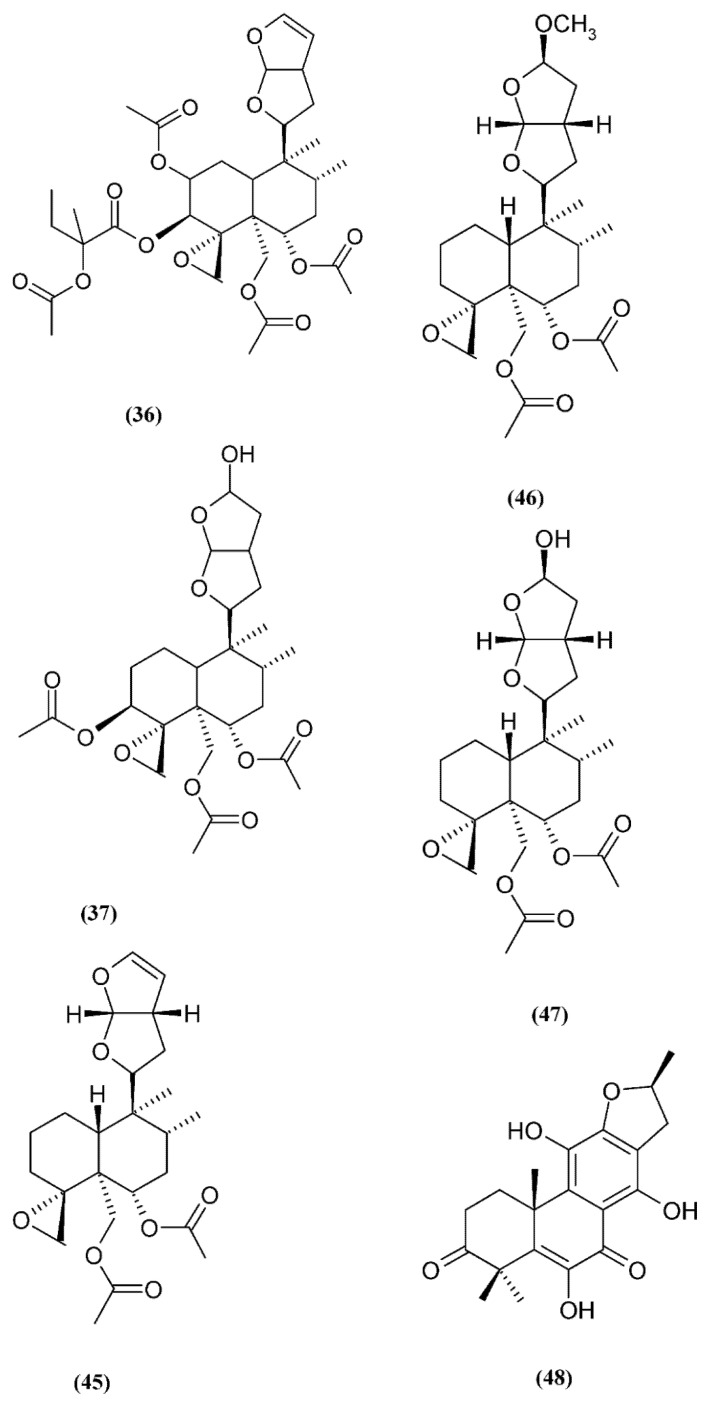

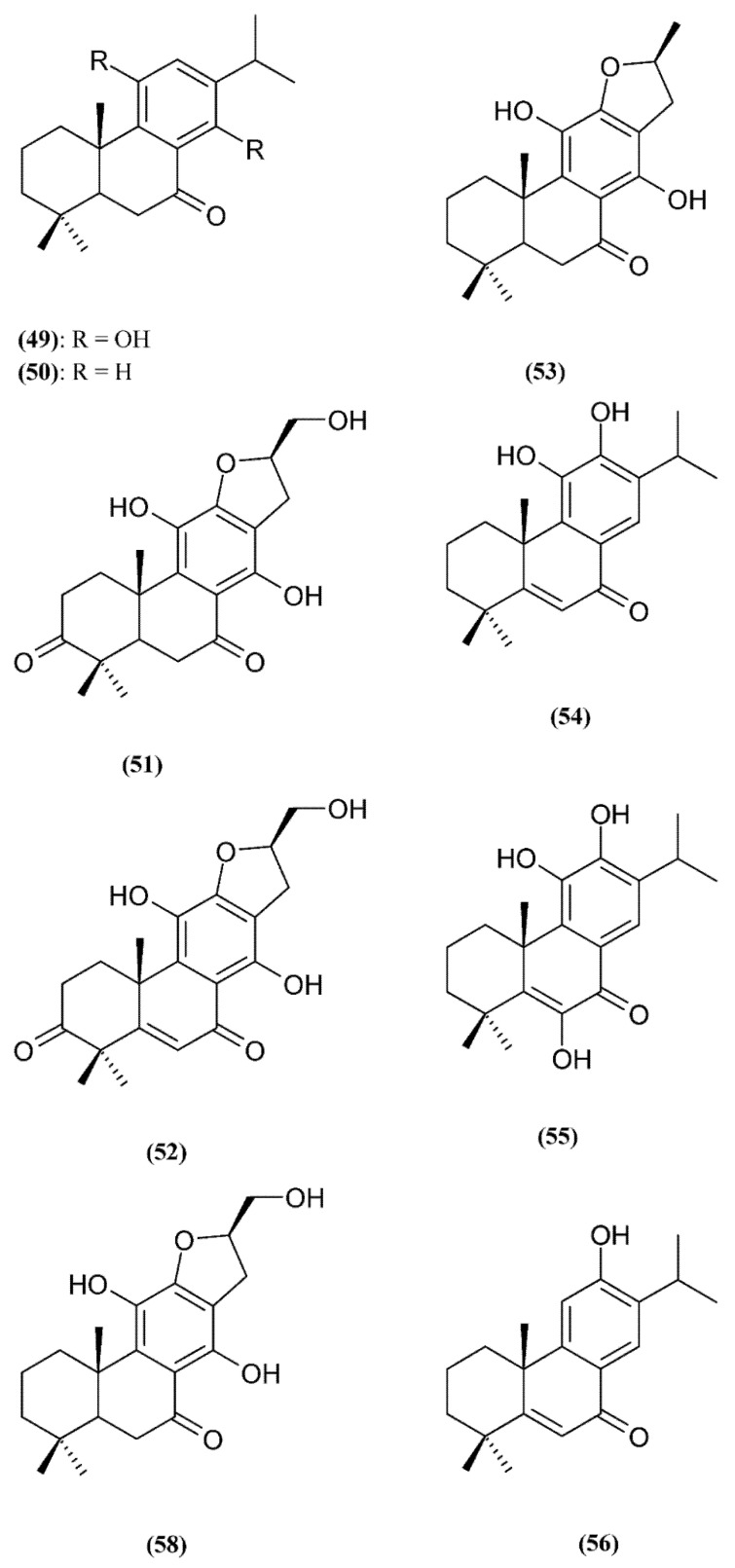

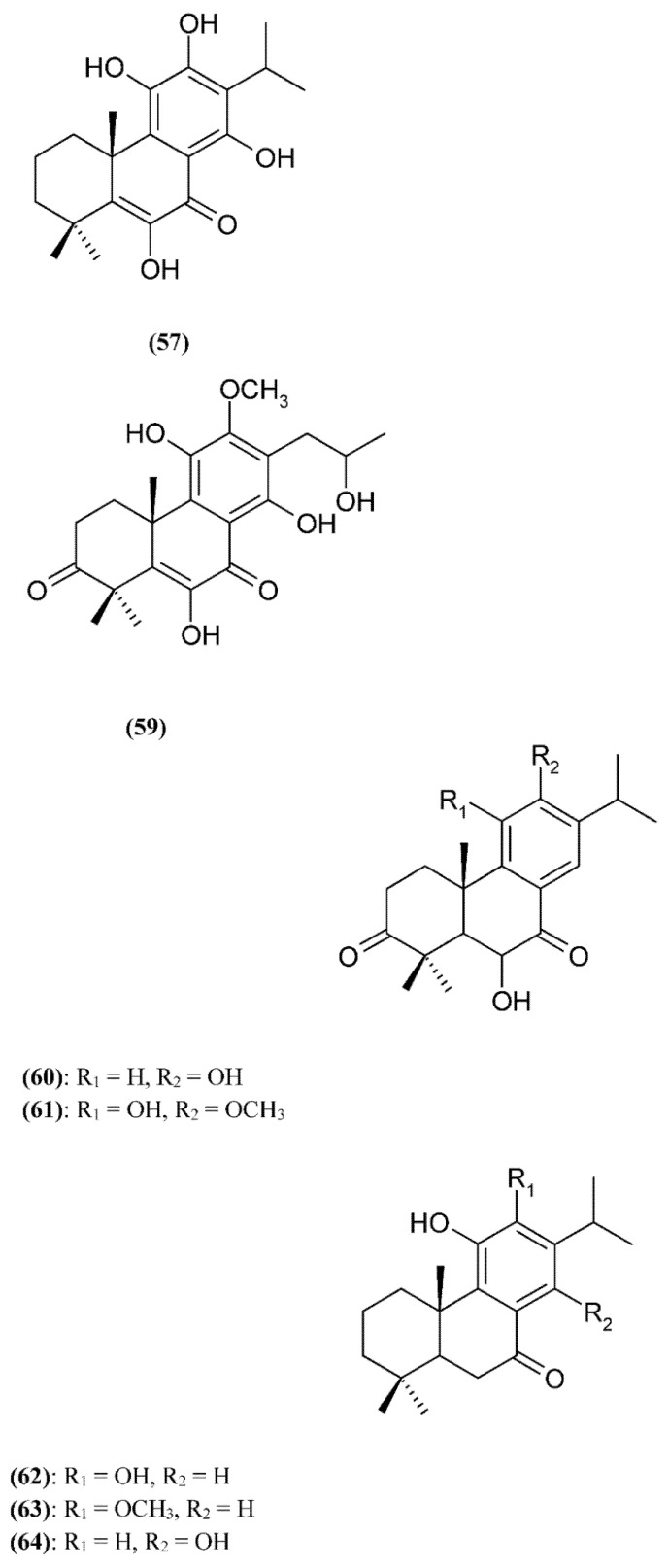

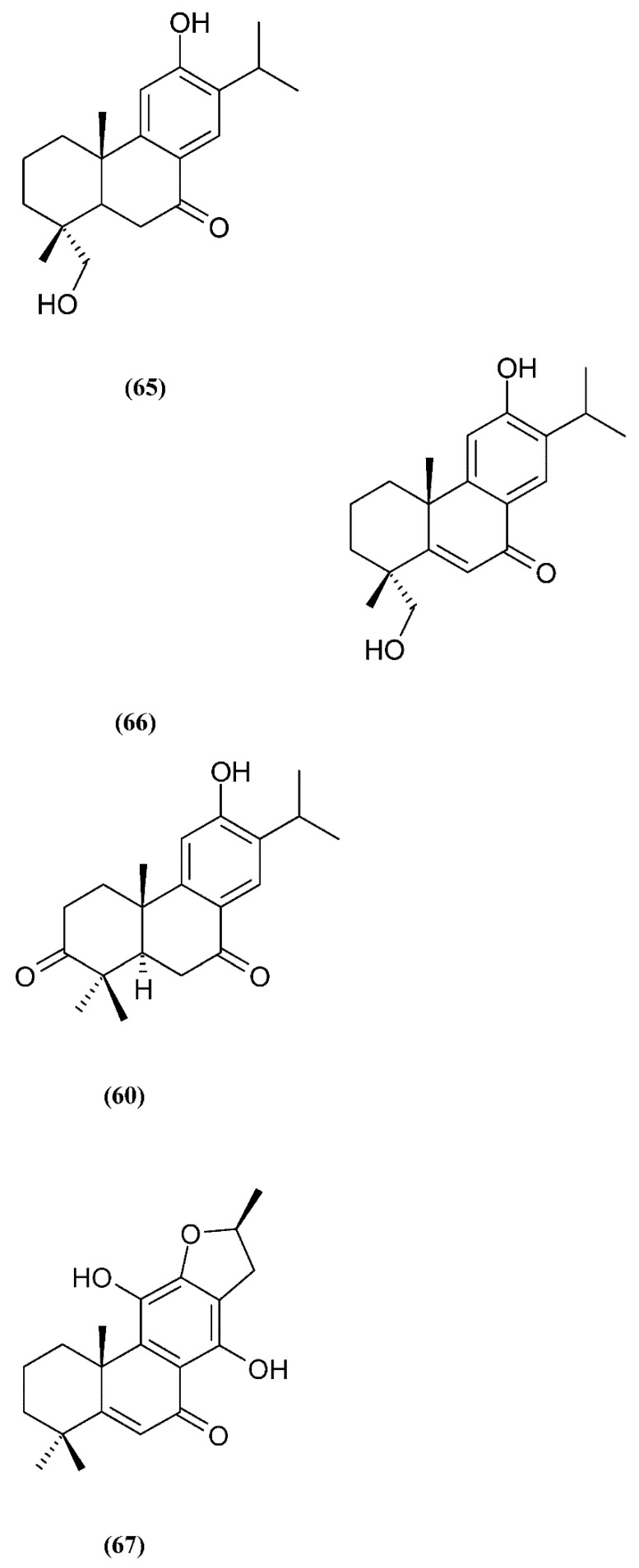

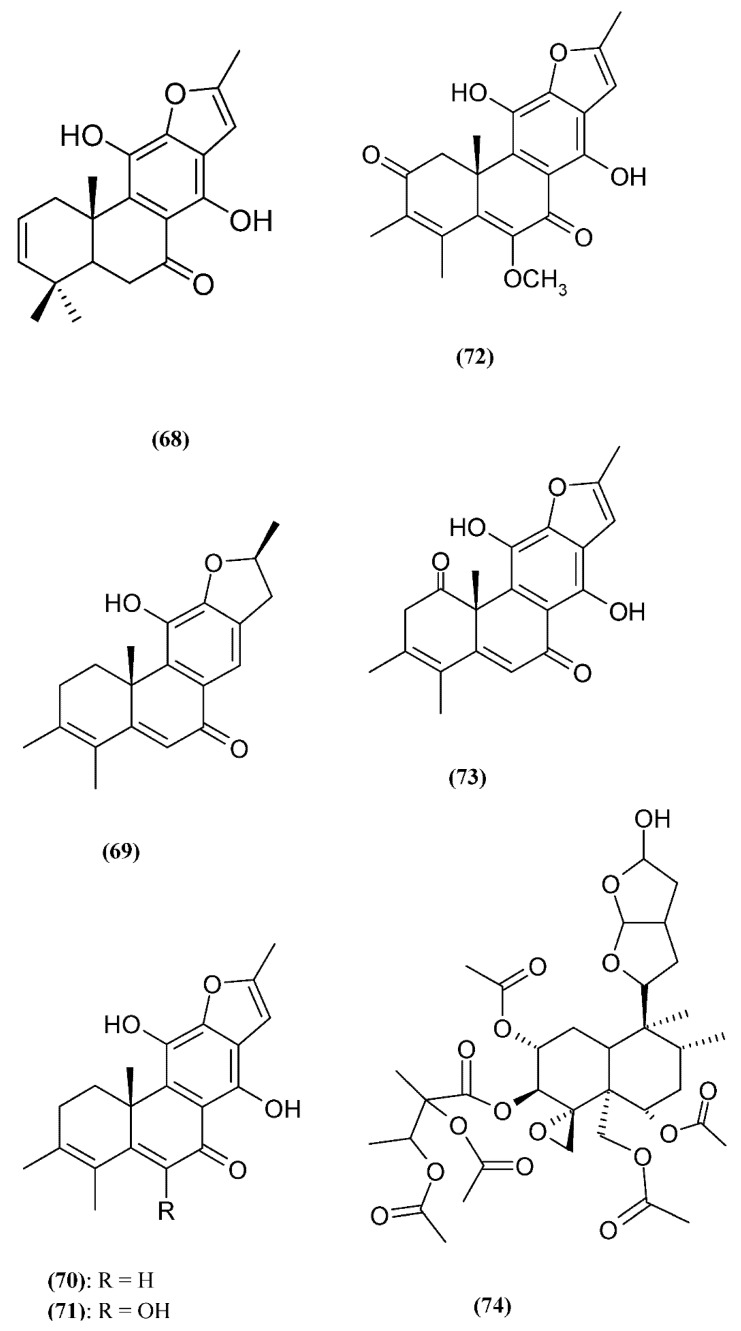

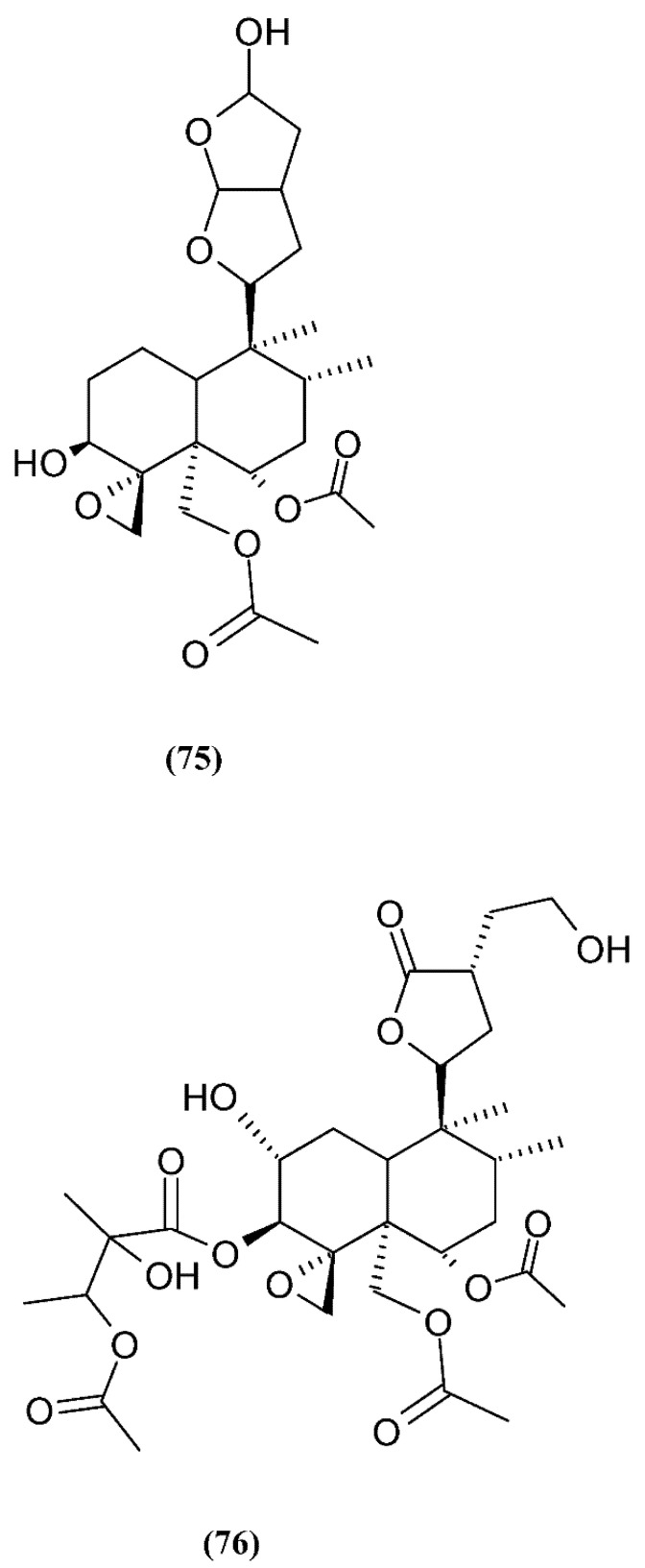

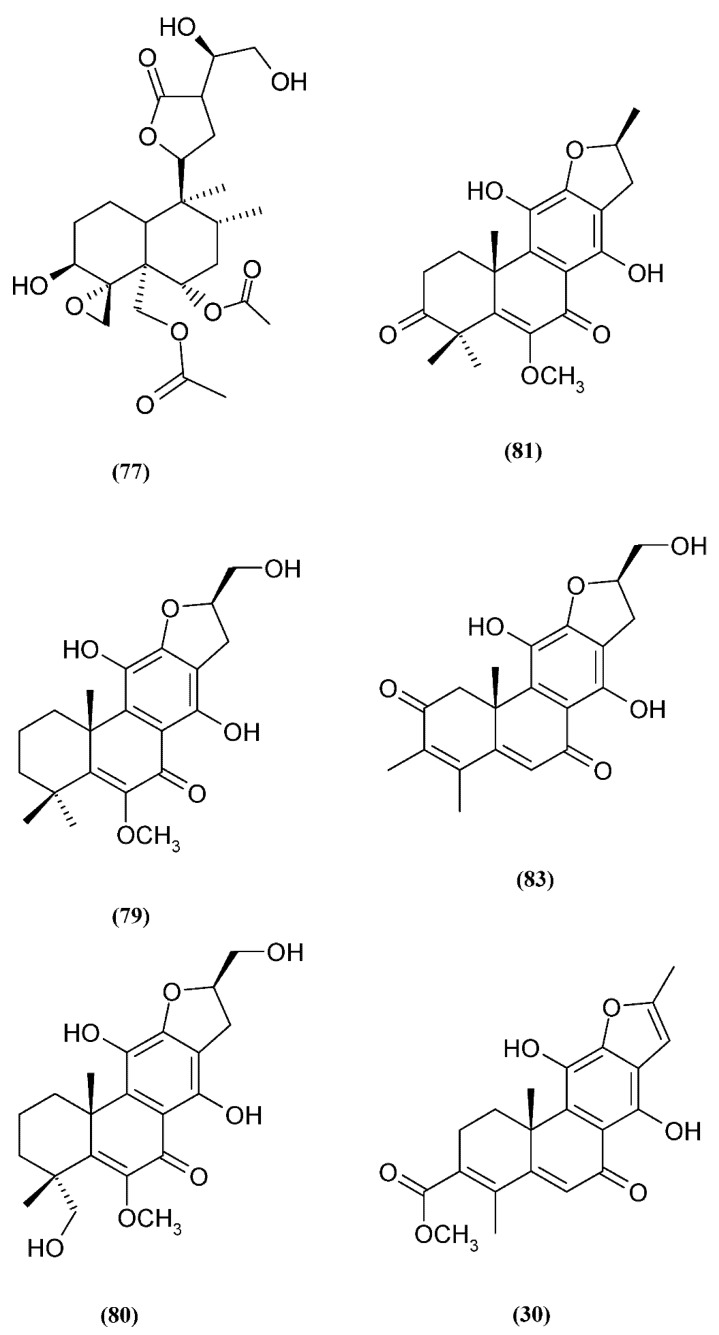

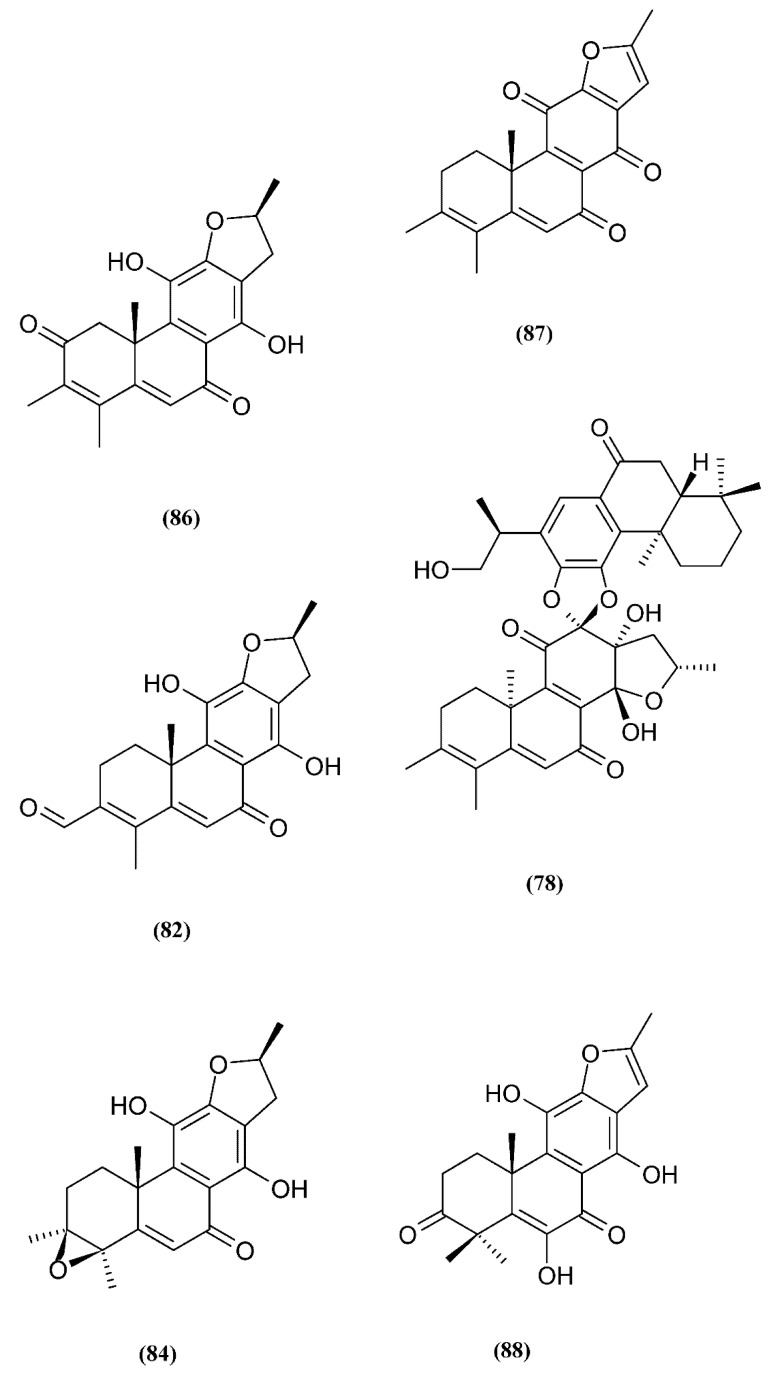

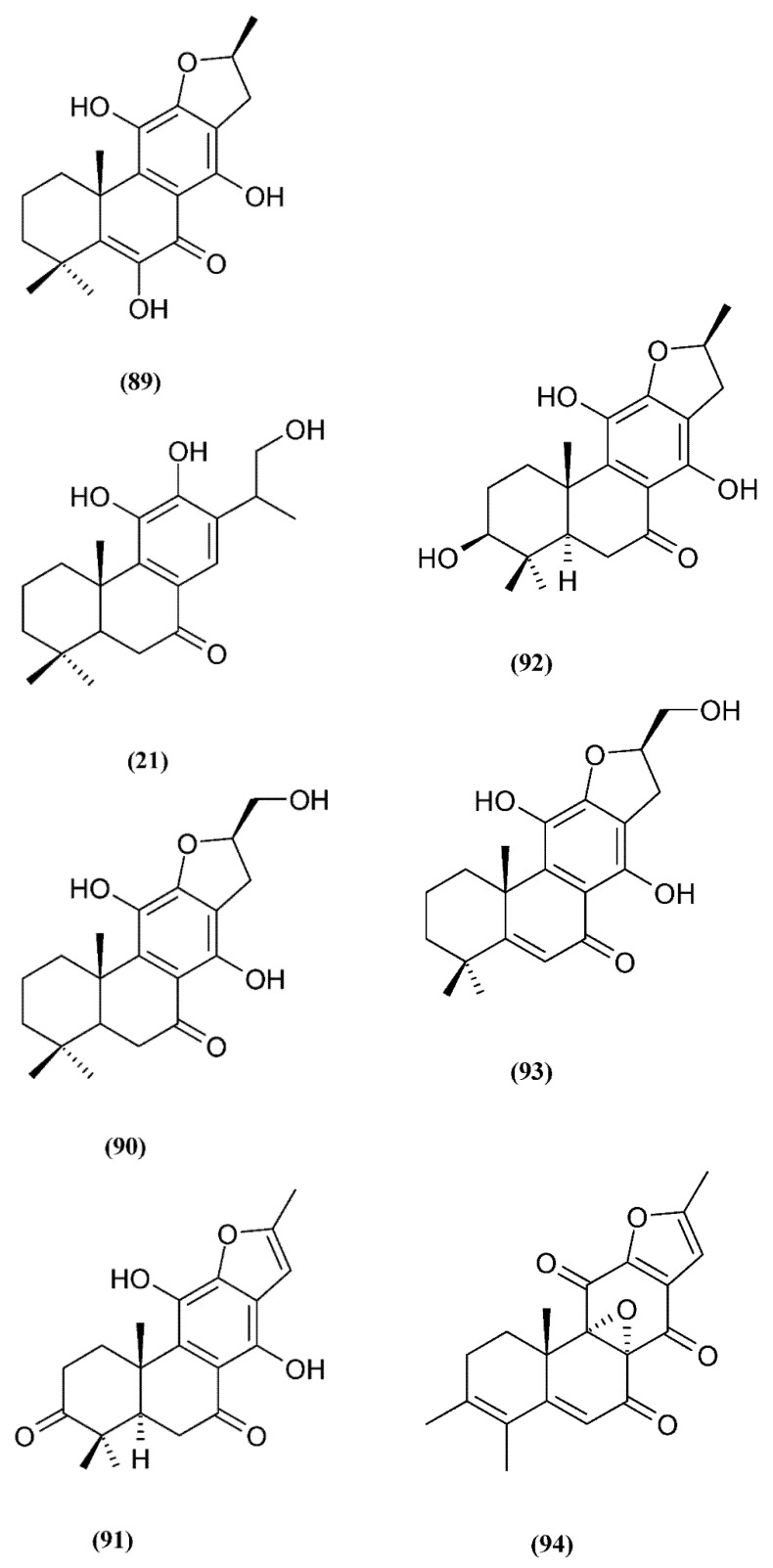

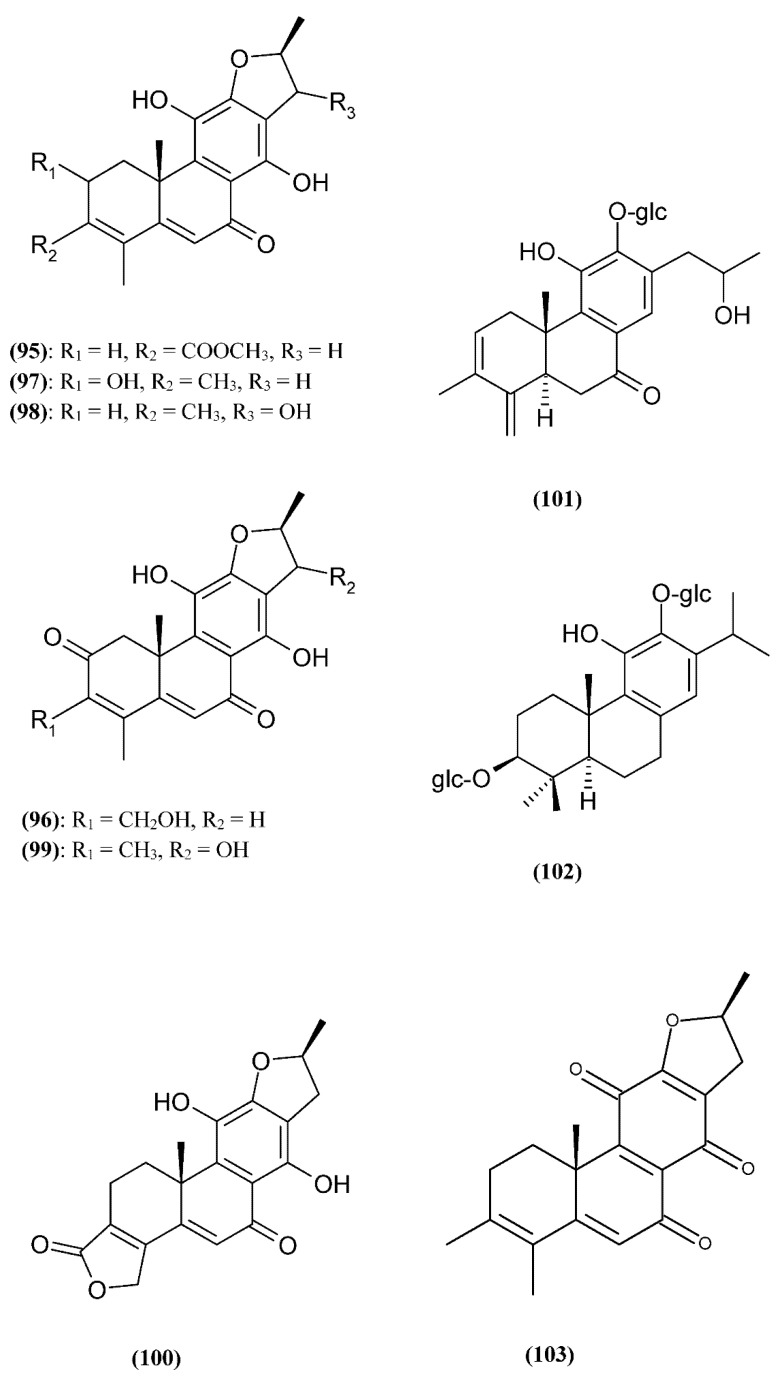

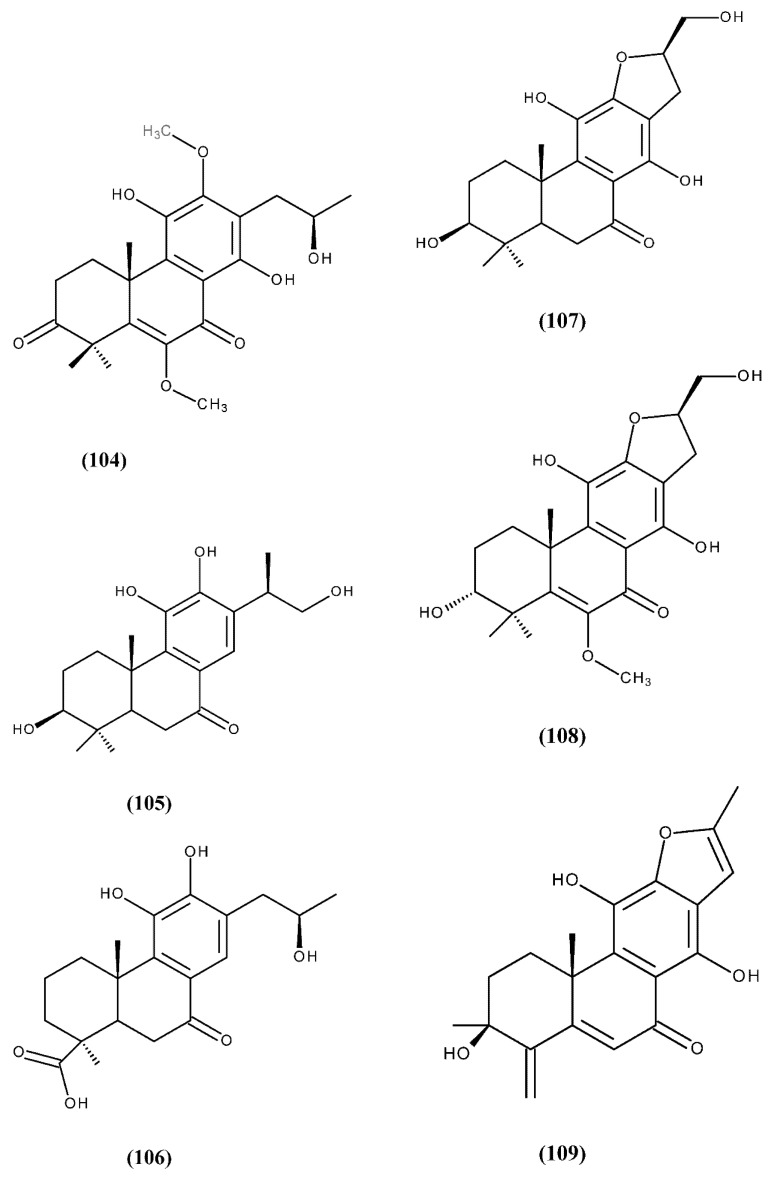

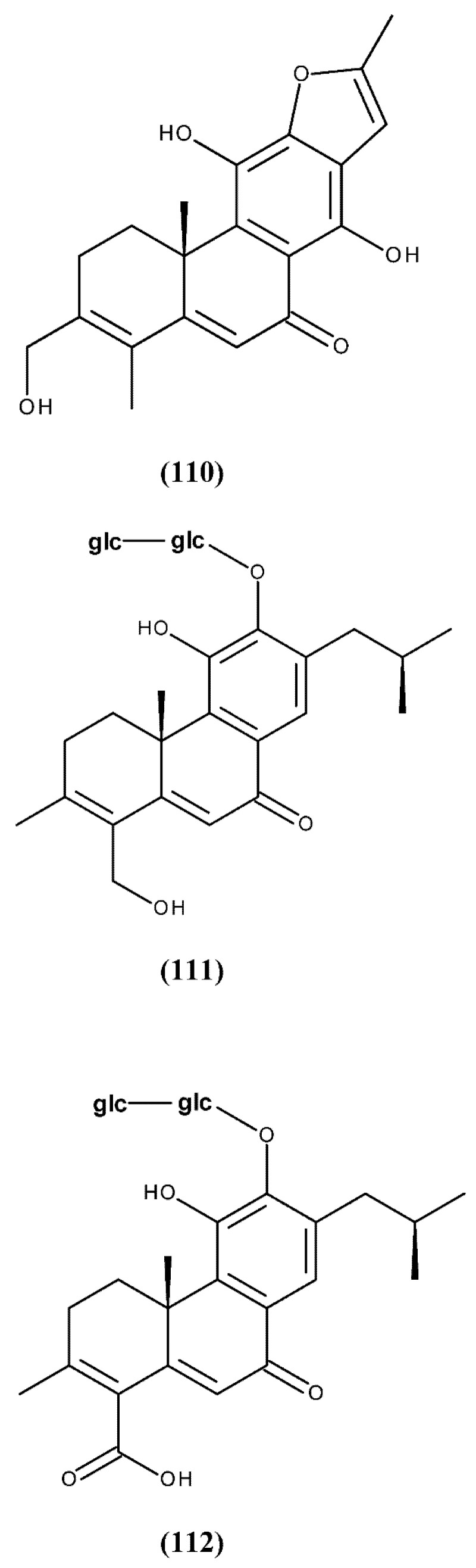
The chemical structure of the compounds isolated from *Clerodendrum* genus.

Therefore, this review examines the members of the genus *Clerodendrum* and their diterpenoid presence, highlighting their biological potential in the area of the most often studied activities, such as cytotoxic, antibacterial, antifungal and others. The chemical structures of the constituents are shown in Figure 2. The present review encompasses the literature data describing the diterpenes present in *Clerodendrum* from 1981 to 2022. The main sources of literature data were Google Scholar, Google, PUBS ACS, ScienceDirect, Springer, Ebsco and others.

## 2. A Review of Diterpenoid Compounds Isolated from *Clerodendrum* Genus

Diterpenoids demonstrate various chemical properties, with a variety of polarities, affinity for the organic phase and solubility. Therefore, the solvent and extraction method must be chosen carefully to optimise the extraction process. The selection of solvent not only depends on the plant species, but also on the organ (overground, underground), and the amount of contaminants, including the presence of chlorophyll. The various solvents and methods used for diterpenoid extraction from *Clerodendrum* are given in Tables 1–13, together with the parts of the plants used for isolation.

### 2.1. Clerodendrum bungei

In Chinese folk medicine, *C. bungei* (eng. name: rose glory bower, Chinese name: Chou mu dan) is a plant raw material whose roots and leaves are used to treat boils, haemorrhoids, hypertension, lung cancer and eczema [34]. This species is known to be rich in diterpenoids, some of which exhibit potential biological activities [34,35,36]. The parts of the plant and the extraction methods used for the phytochemical analyses for this plant species are shown in Table 1.

This plant species was first investigated for diterpenoid content by Fan et al. in 1999 [35]. The authors isolated two new royleanone-type compounds from *C. bungei* roots: 9,10-dihydro-3,4,9-trimethyl phenanthro [3,2-b]pyran (7H)-7, 12(8H)-dione (bungone A **(1)**) and 9,10-dihydro-8-hydroxymethyl-3,4,9-trimethylphenanthro [3,2-β]pyran(2H)-7,12-dione (bungone B **(2)**). While these abietane diterpenoids are structurally similar to the royleanones, with both possessing an 11,14-para benzoquinone group, the C-12 has an oxygen enclosed by an additional aliphatic ring instead of a hydroxyl group, which is typical for roylanones. Due to their cytotoxic activities, these compounds are very interesting for further research; like other diterpenes, including horminone or acetyl-horminone, royleanone is able to damage DNA and inhibit topoisomerase I and II [31,40,41].

Liu et al. (2008) [36] isolated other abietane-type diterpenoids from the roots of *Clerodendrum bungei*. Five were new structures: 12-*O*-*β*-D-glucopyranosyl-3,11,16-trihydroxyabieta-8,11,13-triene **(3)**, 3,12-*O*-*β*-D-diglucopyranosyl-11,16-dihydroxyabieta-8,11,13-triene **(4)**, 19-*O*-*β*-D-carboxyglucopyranosyl-12-*O*-*β*-D-glucopyranosyl-11,16-dihydroxyabieta-8,11,13-triene **(5)**, 11,16-dihydroxy-12-*O*-*β*-D-glucopyranosyl-17(15→16),18(4→3)-*abeo*-4-carboxy-3,8,11,13-abietatetraen-7-one **(6)** and 19-hydroxyteuvincenone F **(7)**. All are glycosides, apart from compound **(7)**, and all contain aglycone, either as an abietatriene or abietatetraene.

In addition, the diterpenoids ajugaside A **(8)**, uncinatone **(9)** and teuvincenone F **(10)**, first isolated from other plant materials, were also isolated, purified and identified from the aqueous acetone crude extract of *C. bungei* roots. The identified compounds were tested for their potential cytotoxic activity against three cell lines: B16 murine melanoma, HGC-27 human gastric, and HEK-293 human epithelial kidney. Of the tested compounds, only uncinatone **(9**), a rearranged abietane derivative containing a 17(15→16), 18(4→3)-*diabeo*-abietane framework, was found to demonstrate moderate cytotoxicity against tested cell lines: the IC_50_ value ranged from 1.2 to 6.4 µM depending on the treated cell line, as indicated by MTT, i.e., 3-(4,5-dimethylythiazol-2-yl)-2,5-diphenyl-2*H*-tetrazolium bromide. This diterpenoid also inhibited cell proliferation and induced cell-cycle G2/M phase arrest [36].

In addition, 12-*O*-*β*-D-glucopyranosyl-3,11,16-trihydroxyabieta-8,11,13-triene **(3)**, 3,12-*O*-*β*-D-diglucopyranosyl-11,16-dihydroxyabieta- 8,11,13-triene **(4)**, ajugaside A **(8)**, uncinatone **(9)** and 19-hydroxyteuvincenone F **(7)** demonstrated significant anti-complement activity on the classical pathway complement system, as expressed by total hemolytic activity [37]. The inhibitory activity of these compounds against the complement system recorded an IC_50_ range from 24 µM to 232 µM. The most active compound was found to be 12-*O*-*β*-D-glucopyranosyl-3,11,16-trihydroxyabieta-8,11,13-triene **(3)** [37]. Kim et al. (2010) postulate that the hydroxyl group in position 3 of this compound may play an important role in its high anti-complement activity. Other diterpenes with glucose, methyl, or hydrogen moieties at position 3 demonstrated significantly lower anti-complement activities [37]. In addition, another two new diterpenoids were isolated from *C. bungei*: 3*β*-(*β*-D-glucopyranosyl)isopimara-7,15-diene-11α,12α-diol **(11)** and 16-*O*-*β*-D- D-glucopyranosyl-3 *β*-20-epoxy-3-hydroxyabieta-8,11,13-triene **(12)** together with other known compounds, such as 12-*O*-*β*-D-glucopyranosyl-3,11,16-trihydroxyabieta-8,11,13-triene **(3)** and 3,12-*O*-*β*-D-diglucopyranosyl-11,16-dihydroxy-abieta-8,11,13-triene **(4)** [34]. All isolated, purified and identified secondary metabolites were evaluated for cytotoxicity against the following tumour cell lines: B16 murine melanoma, HGC-27 human gastric and BEL-7402 human hepatocellular carcinoma. Sun et al. (2014) report that only 16-*O*-*β*-D-glucopyranosyl-3*β*-20-epoxy-3-hydroxyabieta-8,11,13-triene **(12)** appeared to be active among all tested compounds; it demonstrated moderate cytotoxicity against B16, HGC-27, and BEL-7402 cells, manifested with IC_50_ values of 8.8, 9.8, and 7.1 µM, respectively [34]. The authors emphasise the structural similarities between this diterpenoid and bioactive compounds isolated from the same plant material [34,35,37]. It is worth adding, that this metabolite has a hydroxyl group at the third carbon, which is believed to be responsible for the biological activities of compounds isolated from *C. bungei* roots [37].

Further studies on *C. bundei* resulted in the isolation and identification of the following diterpenoids: bungnate A **(13)** (12,16-epoxy-6-methoxy-11,14-dihydroxy-17(15→16)-abeo-5,8,11,13,15-abietapentaen-7-one-17-carboxylate), bungnate B **(14)** (19-O-β-D-carboxyglucopyranosyl-11,12,16-trihydroxy-abieta-8,11,13-triene-7-one), 15-dehydrocyrtophyllone A **(15)** (12,16-epoxy-6-methoxy-11,14-dihydroxy-17(15→16)-abeo-5,8,11,13,15-abietapentaen-7-one) 15-dehydro-17-hydroxycyrtophyllone A **(16)** (12,16-epoxy-6-methoxy-11,14,17-trihydroxy-17(15→16)-abeo-5,8,11,13,15-abietapentaen-7-one), and cyrtophyllone A **(17)** [38]. Of these, 15-dehydrocyrtophyllone A **(15)** demonstrated ACE (Angiotensin Converting Enzyme) inhibition activity, with an IC_50_ value of 42.7 µM. Among the tested diterpenoids, none inhibited α-glucosidase [38].

### 2.2. Clerodendrum cyrtophyllum

This genus, known in Chinese medicine as “Da quing”, is recommended for treating infectious diseases, common cold and malaria [42]. Many relevant compounds have been extracted from the plant, including the diterpenoids teuvincenone F **(10)**, uncinatone **(9)** and sugiol **(18)**, the triterpenoids friedelin **(19)** and clerodolone **(20)** and the phytosteroids stigmasta-5,22,25-trien-3*β*-ol and clerosterol. In addition, two new abietane derivatives, cyrtophyllone A **(17)** (16(*S*)-12,16-epoxy-11,13-dihydroxy-6-methoxy-17(15-16)-*abeo*-abieta-5,8,11,13-tetraen-7-one) and cyrtophyllone B **(21)** ((+)-11,12,16-trihydroxy-abieta-8,11,13-trien-7-one) have been isolated from ethanolic extract of the entire *C. cyrtophyllum* plant following cleaning by water and chloroform mix [42]. The former has a 17(15-16)-*abeo*-abietane framework.

The diterpenes sugiol **(18)**, uncinatone **(9)** and cyrtophyllone B **(21)**, also isolated from *C. cyrtophyllum*, have also been identified in *Aegiphila lhotzkyan* roots. These phytocompounds were tested for antiproliferative activity against leukaemia (CEM and HL-60), breast (MCF-7), colon (HCT-8) and skin (B-16) cancer cell lines in three independent experiments [43]. Of these, only cyrtophyllone B **(21)** is able to inhibit the proliferation of all tested tumour cell lines; however, it did not demonstrate strong inhibition (IC_50_ values above 1 µg mL^−1^) [43]. In addition, diterpenoids isolated from *Caryopteris mongolica* roots were found to inhibit acethyl- and butyrylcholineesterase (AChE and BChE) [44]. The extraction method used for the phytochemical analyses of this plant species is shown in Table 2.

### 2.3. Clerodendrum eriophyllum

This unusual plant was previously used in malaria treatment in Kenya [45]. An alcoholic *C. eriophyllum* root bark extract demonstrated significant chemosuppressive properties against *Plasmodium berghei* in infected experimental mice [46]. The first phytochemical study of *Clerodendrum eriophyllum* was recorded by Machumi et. al. in 2010 [47]. The extraction methods used for the phytochemical analyses of this plant species are shown in Table 3. The dichloromethane-methanolic root extract was found to contain ten abietane diterpenoids, with one being a new discovery: 12-hydroxy-8,12-abietadiene-3,11,14-trione **(22)**. The remaining nine diterpenes had previously been isolated from other plant materials: royleanone **(23)**, taxodione **(24)**, 6-deoxy-taxodione **(25)** (11-hydroxy-7,9(11),13-abietatrien-12-one), sugiol **(18)**, ferruginol **(26)**, 6-hydroxysalvinolone **(27)**, 6,11,12,16-tetrahydroxy-5,8,11,13-abietatetra-en-7-one **(28)**, uncinatone **(9)** and 11-hydroxy-8,11,13-abietatriene-12-O-β-xylopyranoside **(29)** [47].

One of the abietane diterpenoids, royleanone **(23)**, was first isolated from *Inula royleana* roots [48]. However, its presence has also been confirmed in other plant species, e.g., in transformed *Salvia austriaca* roots [49] and non-transformed *Salvia officinalis* roots [32]. Royleanone **(23)**, the diterpenoid characterised by the presence of a *p*-quinone grouping in the C ring, is also well known for its various biological activities. It has been found to demonstrate cytotoxicity against the cancer cell lines HeLa and Hep-2, particularly against Hep-2, with an IC_50_ value of 34 µg mL^−1^ [50]. It has also been found to demonstrate some antibacterial activity, but with weaker activity against methycyllin- and vancomycin-resistant *S. aureus* strains (MRSA and VRE) compared to other diterpenoids from outside the *Clerodendrum* genus (MIC = 32 and above 64 µg mL^−1^, respectively) [51].

Taxodione **(24)** is a very well-known abietane-type diterpenoid with a metide-quinone moiety, which was first isolated from entire *Taxodium distichum* plant [52]. This compound has been found to demonstrate in vivo cytotoxic activity against Walker intramuscular carcinosarcoma 256 in rats and in vitro activity against human nosopharynx carcinoma cells KB [52]. Its high cytotoxicity was confirmed in further studies on Hep-2 and HeLa [50] and A549 [30]. This compound also demonstrates weak AChE and BChE inhibition. Computer modelling found the phytocompound to demonstrate low cardio- and genotoxicity and good permeability of the blood–brain barrier [30]. It has also been found to demonstrate strong antibacterial activity, particularly against MRSA and VRE strains (MIC = 4–10 µg mL^−1^) [51].

6-deoxy-taxodione **(25)**, isolated from *C. eriophyllum* roots, is also detected in various parts of other plant species, e.g., in winter cones of *Taxodium distichum* and fruits of *Cupressus sempervirens* [52,53,54]. Like taxodione **(24)**, both isolated from *Cupressus sempervirens* cones, this compound demonstrates potent anti-leishmanial activity, with IC_50_ values of 0.077 µg mL^−1^ for 6-deoxy-taxodione **(25)** and 0.025 µg mL^−1^ for taxodione **(24)**. The two diterpenoids demonstrated much stronger activity against *Leishmania donovani* and its promastigotes than the anti-leishmanial drugs used as controls: pentamidine (IC_50_ 1.62 µg mL^−1^) and amphotericin B (IC_50_ 0.11 µg mL^−1^) [53]. In addition, 6-deoxy-taxodione **(25)** was found to demonstrate potent antibacterial activities against methicillin-resistant *Staphylococcus aureus* (MRSA), with IC_50_ values being 0.80 μg mL^−1^ for **(25)** and 0.85 μg mL^−1^ for **(26)** [53].

Another abietane-type diterpenoid is sugiol **(18)**, isolated from *Clerodendrum eriophyllum* roots. This compound has an oxygen atom connected to the B ring and an aromatic C ring. This unusual aromatic diterpene demonstrates various antioxidant, antibacterial, antiviral, anticancer, anti-tumour and anti-inflammatory activities [55]. Its antioxidant activity is similar to those of α-tocopherol and ascorbic acid based on DPPH (2,2-diphenyl-1-picrylhydrazyl) radical scavenging assay (84% and 82%, respectively) [56]. Sugiol **(18)** also demonstrates a concentration-dependent inhibitory effect (72.4%) against NO (nitric oxide), at a concentration of 100 μg mL^−1^; it also demonstrated similar superoxide radical scavenging activity at a concentration of 250 μg mL^−1^, to ascorbic acid and α-tocopherol activities (73% for sugiol compared to 73% and 74.5%, respectively) [56]. Sugiol **(18)** is also active against various foodborne pathogenic bacteria but neutralises Gram-positive bacteria more effectively than Gram-negative bacteria. When isolated from *Metasequoia glyptostroboides* cones, the compound was also found to demonstrate stronger antibacterial action against Gram-positive bacteria than the streptomycin used as a control [57]. Sugiol **(18)** has also been found to exhibit antiviral activity against the H1N1 virus in infected Madin-Darby canine kidney (MDCK) cells: no cytopathic changes were observed following 72 h of exposure following treatment with 500 μg mL^−1^ sugiol **(18)**. Hence, sugiol **(18)** could be a potential antiviral compound that can prevent H1N1-mediated cytopathy in MDCK cells [58].

The diterpenoid sugiol **(18)** also demonstrated cytotoxic activity against tumour cell lines, inhibiting the growth of three prostate tumour cell lines (LNCap, PC3 and DU145) and a non-tumorigenic cell line (MCF10A) [55]. Similarly, sugiol **(18)** treatment was found to reduce tumour weight and volume by as much as 75% in mice subcutaneously injected with DU145 cells in comparison with the control group. However, sugiol **(18)** did not affect the body weight of the mouse [55].

The abietane diterpenoid ferruginol **(26)** was first isolated in 1939 from the *Podocarpus ferruginea* tree. Structurally, ferruginol is similar to sugiol **(18)**, although it lacks an oxygen in the B ring. The biologically active ferruginol has been recorded in many plants including those of the Podocarpaceae, Cupressaceae, Lamiaceae and Verbenaceae [59]. This diterpenoid exhibits antibacterial and antifungal activities [60]. It has been found to inhibit the growth of *Bacillus brevis*, *B. subtilis* and *Staphylococcus aureus*, with inhibition zone diameters of 18, 10 and 9 mm, respectively. Ferruginol **(26)** demonstrated fungicidal activity against the pathogenic *Paecilomyces variotii*, with an inhibition zone of 10 mm [60], and Ferruginol **(26)** isolated from *Chamaecyparis lawsoniana* cones also demonstrated antibacterial activity against *S. aureus*, with MIC values ranging from 4 to 16 μg mL^−1^ depending on the strain [61]. It has demonstrated potent antimalarial activity [59], with EC_50_ values against *Plasmodium falciparum* ranging from 2.47 to 19.57 μM, depending on the strain [59]. In addition, ferruginol **(26)** has displayed moderate cytotoxic activity against NALM-6 human leukaemia lymphoblastic cells (IC_50_ 27.2 μg mL^−1^) and promyelocytic HL-60 cells (IC_50_ 33.6 μg mL^−1^) [62].

The abietane diterpenoid 6-hydroxysalvinolone **(27),** containing oxygen and hydroxyl groups in the B ring, demonstrates strong cytotoxicity against carcinoma cell lines. Following isolation from *Salvia chorassanica* roots, the compound exhibited strong cytotoxic activity against HL-60 and K562 cell lines with IC_50_ values of 36.3 and 33.3 μM, respectively. It appeared to demonstrate a substantially less cytotoxic effect on non-cancerous human cell lines. When administered at concentrations of 2.5 and 5.0 μM for 48 h, it also enhanced the expression of the proapoptotic protein Bax, and cleaved caspase-3 and PARP [63]. It also was found to exhibit moderate cytotoxic activity against monkey kidney fibroblasts (VERO) with an IC_50_ level of 4.5 μg mL^−1^ [47]. Similarly to taxodione **(24)**, 6-hydroxysalvinolone **(27)** also demonstrated antifungal activity, especially against *Candida neoformans* with an IC_50_ value of 0.96 μg mL^−1^. In the same assay, the IC_50_ of taxodione **(24)** was found to be 0.58 μg mL^−1^, which is comparable with that of standard amphotericin B (IC_50_ = 0.44 μg mL^−1^) [47].

Another abietane-type diterpenoid is 6,11,12,16-tetrahydroxy-5,8,11,13-abietatetra-en-7-one **(28)**, isolated from *Avicennia marina* twigs; it differs from 6-hydroxysalvinolone **(27)** by the presence of a hydroxyl group in the isopropyl moiety. It demonstrated moderate antiproliferative properties against L-929 (mouse fibroblasts) and K562 (human chronic myeloid leukaemia), and cytotoxic activities against the HeLa (human cervix carcinoma) cell line [64]. In biological tests, 6,11,12,16-tetrahydroxy-5,8,11,13-abietatetra-en-7-one **(28)** demonstrated GI_50_ (concentration causing 50% cell growth inhibition) values of 9.6 and 8.9 μg mL^−1^, against L-929 (DSM ACC 2, mouse fibroblasts) and K562 cell lines (DSM ACC 10, human chronic myeloid leukaemia), and a CC_50_ (concentration that reduced the cell viability by 50%) of 18 μg mL^−1^ against the HeLa cell line [64]. The compound also demonstrated antibacterial activity against Gram-positive and Gram-negative bacteria and antifungal potential. A study of its antibacterial activity against *Bacillus subtilis* ATTC 6 633 (IMET) NA, *Bacillus subtilis* ATTC 6 633 (IMET) AS, *Escherichia coli* SG 458, *Pseudomonas aeruginosa* K 799/61, *Mycobacterium vaccae* IMET 10 670, *Sporobolomyces salmonicolor* SBUG 549, *Candida albicans* BMSY 212 and *Penicillium notatum* JP [64] found zone inhibition to range from 12 mm (for *C. albicans*) to 25 mm (for *B. subtilis* ATTC 6 633 (IMET) AS) [64].

Uncinatone **(9)**, a diterpenoid known for its biological activity, also exhibits potent antileishmanial activity. The IC_50_ value for *L. donovani* is 0.2 μg mL^−1^ [47].

### 2.4. C. formicarum

The *abeo*-abietane diterpenoid formidiol **(30)** was first obtained by methanolic extraction of *Clerodendrum formicarum* leaves and chromatographic separation of its triterpenoid constituents [65]. It was accompanied by the diterpenoid 12,16-epoxy-11,14-dihydroxy-6-methoxy-17(15→16)-abeo-abieta-5,8,11,13,15-pentanene-3,7-dione **(31)**, which had been previously isolated from a hexane extract of *Aegiphila lhotzkiana* roots. It was found to demonstrate antiproliferative activity against the leukaemia cell lines HL-60 (IC_50_ 4.4 μM) and CEM (IC_50_ 8.4 μM) [43]. Due to its structural similarity to formidiol **(30)**, compound **(31)** should be included in future studies of anti-proliferative activity. The extraction method used for the phytochemical analyses of this plant species is shown in Table 4.

### 2.5. Clerodendrum inerme

Studies on the aerial parts of *Clerodendrum inerme* resulted in the isolation of cleroinermin **(32)** a *neo*-clerodane diterpenoid [66] consisting of a bicyclic ring decalin moiety and a six-carbon side chain including a furane skeleton. The compound, first isolated from *Heteroplexis micocephala*, showed neuroprotective activity against MPP+ induced PC12-syn cell damage, with a relative cell proliferation rate of 104.32% [67]. Elsewhere, the *neo*-clerodane diterpenoids clerodendrin B **(33)**, 3-epicaryoptin **(34)**, clerodendrin C **(35)**, 2-acetoxyclerodendrin B **(36)** and 15-hydroxyepicaryoptin **(37)** have since been isolated [68]. The extraction methods used for the phytochemical analyses of this plant species are given in Table 5. These compounds have been found to be effective antifeedants against *Earias vitella* at 10 µg cm^−3^ of diet (30 µg g^−1^) and against *Spodoptera litura* at 10 µg cm^−2^ of leaf mass [68].

*C. inerme* has become an interesting subject of research for diterpenoid isolation. The aerial parts are a source of the neo-clerodane-type diterpenoids: clerodermic acid **(38)**, inermes A **(39)** and B **(40)**, as well as 14,15-dihydro-15β-methoxy-3-epicaryoptin **(41)** [69,71]. Among these compounds, clerodermic acid **(38)** deserves special attention due to its strong biological activity. The compound, isolated from the dichloromethane extract of the aerial part of *Salvia nemorosa*, was found to reduce the viability of A549 cells in a concentration-dependent manner, with an IC_50_ of 35 µg mL^−1^ at 48 h, based on the MTT assay [72]. Furthermore, clerodermic acid treatment resulted in various morphological changes, including diminished cell density, membrane blebbing and an increased number of floating cells, all of them being a manifestation of cell death **(38)**. DNA ladder, DAPI staining, cell cycle analysis, and annexin V/PI testing indicated that clerodermic acid demonstrates strong geno- and cytotoxicity and is able to induce apoptosis in A549 cells, as evidenced also by DNA fragmentation and chromatin condensation [72].

*C. inerme* aerial parts have also been found to include a newly rearranged abietane diterpenoid, crolerodendrum B **(42)**, as well as other known diterpenoids, such as crolerodendrum A **(43)**, uncinatone **(9)** and harwickiic acid **(44)** [70]. Harwickiic acid **(44)** was first isolated from *Sindora sumatrana* MIQ fruits [73]. This clerodane-type diterpenoid, obtained from the stem bark of *Croton sylvaticus*, was found to demonstrate significant antileishmanial activity against *L. donovani* promastigotes with an IC_50_ of 31.57 µM, as well as cytotoxic activity against RAW 264.7 (CC_50_ = 247.83 μM) [74]. Harwickiic acid **(44)**, isolated from *C. inerme* aerial parts, together with crolerodendrum B **(42)** and uncinatone **(9)** also demonstrates strong antioxidant activity measured as DPPH radical-scavenging activity; these compounds have been found to have respective ED_50_ values of 11.3 µM **(44)**, 17.6 µM **(42)** and 10.1 µM **(9)** [70].

### 2.6. Clerodendrum infortunatum

Crystallization and chromatographic separation of the leaf extract resulted in the isolation and identification of the clerodane diterpenoids clerodin **(45)**, 15-methoxy-14,15-dihydroclerodin **(46)** and 15-hydroxy-14,15-dihyroclerodin **(47)** [75]. The extraction methods used for the phytochemical analyses of this plant species are shown in Table 6. The isolated compounds were tested against *Helicoverpa armigera*. Studies on the growth inhibition potential of these diterpenoids found topical application of clerodin **(45)**, 15-methoxy-14,15-dihydroclerodin **(46)** and 15-hydroxy-14,15-dihyroclerodin **(47)** to yield GI_50_ values of 13, 21 and 11 ppm, respectively; in contrast, azadirachtin was found to have a GI_50_ value of 15 ppm [75].

The purified diterpenoids, together with their extracts and fractions, also demonstrated insecticidal activity against the highly polyphagous cotton bollworm (*Helicoverpa armigera*) [77]. The antifeedant activity of the isolated diterpenoids was tested using choice and no-choice tests with 24- and 48-h observation intervals. In the no-choice test conditions, clerodin **(45)** and 15-methoxy-14,15-dihydroclerodin **(46)** demonstrated significantly higher antifeedant activity compared to high concentration azadirachtin, the key ingredient in many commercial pesticides [77], with the second diterpenoid demonstrating similar antifeedant activity to that of azadirachtin. In the choice test conditions, all isolated and identified compounds, as well as azadirachtin, demonstrated 100% antifeedant activity at the highest concentration. Furthermore, clerodin **(45)** has also been found to demonstrate antifeedant activity against *Earias vitella* and *Spodoptera litura* [68]. The antifeedant index (AI_50_) values for clerodin **(45)**, 15-methoxy-14,15-dihydroclerodin **(46)** and 15-hydroxy-14,15-dihyroclerodin **(47)** were found to be 6, 6, and 8 ppm in the choice tests, and 8, 9, and 11 ppm in the no-choice tests, respectively.

The antifeedant activity of clerodanes has been attributed to the presence of a perhydrofuranofuran moiety and the degree of its unsaturation; a significant role may also be played by the presence of a trans-decalin ring system bearing an epoxide, together with acetate groups [78,79]. These results suggest that the diterpenoids isolated from *Clerodendrum infortunatum* leaf extract offer promise as biopesticides and require further studies [77].

### 2.7. Clerodendrum kaichianum

*Clerodendrum kaichianum* P. S. Hsu is known to be the source of two new abietane-type compounds, *viz.* 17-hydroxyteuvincenone G **(51)** and 17-hydroxyteuvincen-5(6)-enone G **(52)**, as well as four known diterpenoids: teuvincenone A **(48)**, 11,14-dihydroxyabieta-8,11,13-trien-7-one **(49)**, dehydroabietan-7-one **(50)** and sugiol **(18)** [80]. These new secondary metabolites demonstrated relatively strong cytotoxic activities against HL-60 and A-549 cell lines in vitro based on the MTT assay. This action was compared to *cis*-platin, which was used as a control compound. In addition, 17-hydroxyteuvincenone G **(51)** yielded IC_50_ scores of 5.95 and 9.37 µM for HL-60 and A-549 cells, respectively; this activity was slightly higher than that of 17-hydroxyteuvincen-5(6)-enone G **(52)** (IC_50_ of 15.91 and 10.35 µM against the same cell lines) [80].

Further chromatographic separation from *C. kaichianum* stem extract resulted in the isolation of a newly rearranged abietane diterpenoid with five known compounds: villosin A **(53)**, salvinolone **(54)**, 14-deoxyloleon U **(55)**, 5,6-dehydrosugiol **(56)**, and coleon U **(57)**. This new diterpenoid was identified as (16R)-12,16-epoxy-11,14,17-trihydroxy-17(15→16)-abeo-8,11,13-abietatrien-7-one **(58)** [81]. Villosin A **(53)**, salvinolone **(54)** and 5,6-dehydrosugiol **(56)** were noted in the *Clerodendrum* genus for the first time. All extraction methods used for the phytochemical analyses of this plant species are shown in Table 7. All isolated constituents were tested for their cytotoxic activities against the viable HL-60 tumour cell line based on the MTT assay. The highest cytotoxic activity was demonstrated by (16R)-12,16-epoxy-11,14,17-trihydroxy-17(15→16)-abeo-8,11,13-abietatrien-7-one **(58)** with an IC_50_ value of 18.5 µM, with villosin A **(53)** and coleon U **(57)** demonstrating IC_50_ values of 20.1 and 24.1 µM, respectively. Salvinolone **(54)**, 14-deoxyloleon U **(55)** and 5,6-dehydrosugiol **(56)** demonstrated more than two-fold weaker cytotoxic activity, with IC_50_ values over 40 µM [81].

### 2.8. Clerodendrum kiangsiense and C. mandarinorum

A phytochemical study on the aerial parts of *C. kiangsiense* resulted in the isolation of eight diterpenoids, one of which was a novel *abeo*-abietane diterpenoid. Spectroscopic analyses resulted in its identification as 12-methoxy-6,11,14,16-tetrahydroxy-17(15→16)-abeo-5,8,11,13-abietatetraen-3,7-dione **(59)** [82]. The remaining secondary metabolites were identified as mandarone A **(60)** ((5*R*,10*S*)-12-hydroxy-8,11,13-abietatriene-37-dione), taxusabietane A **(61)**, 12-O-demethylcryptojaponol **(62)**, cryptojaponol **(63)**, 11,14-dihydroxy-8,11,13-abietatrien-7-one **(64)**, fortunin E **(65)** and fortunin F **(66)** [82]. Mandarone A **(60)** had previously been isolated from *Clerodendrum mandarinorum* stem [83] and *Euonymus lutchuensis* roots [84].

Various other mandarones have also been isolated from *C. mandarinorum* stem, including mandarone B **(67)** ((16 *S*)-12,16-epoxy-11,14-dihydroxy-17(15→16)-abeo-abieta-5,8,11,13-tetraene-7-one), mandarone C **(68)** (12,16-epoxy-11,14-dihydroxy-17(15→16)-abeo-abieta-2,5,8,11,13,15-hexaene-7-one) [84], mandarone D **(69)** (16*S*)-12,16-epoxy-l1-hydroxy-17(15→16),18(4→3)-diabeo-abieta-3,5.8,11,13-pentaene-7-one, mandarone E **(70)** (12.l6-epoxy-l1,14-dihydroxy-17(15→16),18(4→3)-diabeo-abieta-3,5,8,11,13,15-hexaene-7-one), mandarone F **(71)** (12,16-epoxy-6,11,14-trihydroxy-17(15→16),18(4→3)-diabeo-abieta-3,5,8,11,13,15-hexaene-7-one), mandarone G **(72)** (12,16-epoxp-11,14-dihydroxy-6-methoxy-17(15→16),18(4→3)-diabeo-abieta-3,5,8,11,13,15-hexaene-2,7-dione) and mandarone H **(73)** (12,16-epoxy-11,14-dihydroxy-17(15→16),18(4→3)-diabeo-abieta-3,5,8,11,13,15-hexaene-1,7-dione) [85]. The extraction methods used for the phytochemical analyses of these plant species are shown in Table 8 and Table 9.

Taxusabietane A **(61)**, isolated from bark extract of *Taxus wallichiana* Zucc. (in addition to taxusabietane C and taxamairin F), was found to demonstrate considerable lipoxygenase (LOX) inhibitory activity at an IC_50_ of 57 μM compared to controls (baicalein IC_50_ 22.1 μM) based on in vitro lipooxygenase inhibition assay and in vivo carrageenan-induced paw oedema model [86]. Cryptojaponol **(63)**, isolated from extracted *Taxodium distichum* bark, demonstrated moderate cytotoxic activity against human pancreatic carcinoma (PANC-1) [87] with an EC_50_ of about 38 μM and selective index (SI) of 7.9 [87].

In addition, 11,14-dihydroxy-8,11,13-abietatrien-7-one **(64)**, an abietane diterpenoid found in *Clerodendrum kiangsiense* aerial parts, exhibits some interesting biological activities. Costa-Lotufo et al. (2004) found it to demonstrate moderate cytotoxic activity against tumour cell lines, together with as well as carnasol, isolated from *Hyptis martiusii* roots [88]. Zadali et al. (2020) also reported it to be present in the aerial parts and roots of *Zhumeria majdae* and to show promising antiprotozoal activity; the IC_50_ value was found to be 8.65 μM, with a selectivity index (SI) of 4.6 [89]. Additionally, it has also been found to demonstrate greater binding affinity at the active site of AChE in comparison to donepezil [90].

### 2.9. Clerodendrum splendens

Scientific research on this species allowed to isolate and identify four new clerodane diterpenoids, namely 2α-acetoxy-3β-(2′,3′-diacetoxy-2′-methyl)-butanoyloxy-14-hydro-15-hydroxyclerodin **(74)**, 3β,15-dihydroxy-14-hydro-clerodin **(75)**, 2α,15-dihydroxy-3β-(2′-hydroxy-2′-methyl-3′-acetoxy)-butanoyloxy-6α,18-diacetoxy-4α,17-epoxy-clerodan-11,16-lactone **(76)** and 3β,14S,15-trihydroxy-6α,18-diacetoxy-4α,17-epoxy-clerodan-11,16-lactone **(77)** [91]. The extraction method used for the phytochemical analyses of this plant species is shown in Table 10. Faiella et al. (2013) tested these compounds for their potential antiproliferative activity against HeLa cells. Briefly, the HeLa cells were incubated for 24 h with the diterpenoids at a concentration of 50 μM, and the results were compared with 15 μM phenethylisothiocyanate (PEITC) as a control. The results indicate that 2α-acetoxy-3β-(2′,3′-diacetoxy-2′-methyl)-butanoyloxy-14-hydro-15-hydroxyclerodin **(74)** and 2α,15-dihydroxy-3β-(2′-hydroxy-2′-methyl-3′-acetoxy)-butanoyloxy-6α,18-diacetoxy-4α,17-epoxy-clerodan-11,16-lactone **(76)** exhibit cell growth inhibition activity. In addition, the IC_50_ values for the two compounds, viz., **(76)** and **(74)**, were found to be 101 μM and 98 μM, respectively, after 72 h incubation [91].

### 2.10. Clerodendrum trichotomum

Trichotomone **(78)** was first isolated from *Clerodendrum trichotomum* roots by careful semi-preparative chromatographical analysis. This diterpenoid is a rare phenolic ketal of a regular abietane derivative, cyrtophyllone B **(21)**, and a rearranged abietane derivative related to uncinatone **(9)** [89]. Trichotomone **(78)** demonstrates moderate cytotoxic activity against some tumour cell lines (A549, Jurkat, BGC-823 and 293T WT) with IC_50_ values ranging between 7.51 and 19.38 µM [92].

Wang et al. (2013) report the isolation of various other diterpenoid compounds from the species, including 17(15→16)-*abeo*-abietane (6-methoxyvillosin C **(79)** (=(10*R*,16*R*)-12,16-epoxy-11,14,17-trihydroxy-6-methoxy-17(15→16)-abeoabieta-5,8,11,13-tetraene-7-one), 18-hydroxy-6-methoxyvillosin C **(80)** (=(10*R*,16*R*)-12,16-epoxy-6-methoxy-11,14,17,18-tetrahydroxy-17(15→16)-abeo-abieta-5,8,11,13-tetraene-7-one) and (10*R*,16*S*)-12,16-epoxy-11,14-dihydroxy-6-methoxy-17(15→16)-abeo-abieta-5,8,11,13-tetraene-3,7-dione **(81)** and 17(15→16),18(4→3)-*diabeo*-abietane diterpenoids (trichotomone D **(82)** (=10*R*,16*S*)-12,16-epoxy-11,14-dihydroxy-18-oxo-17(15→16),18(4→3)-diabeo-abieta-3,5,8,11,13-pentaene-7-one, (10*R*,16*R*)-12,16-epoxy-11,14,17-trihydroxy-17(15→16),18(4→3)-diabeo-abieta-3,5,8,11,13-pentaene-2,7-dione **(83)** and trichotomone F **(84)** =(3*S*,4*R*,10*R*,16*S*)-3,4:12,16-diepoxy-11,14-dihydroxy-17(15→16),18(4→3)-*diabeo*-abieta-5,8,11,13-tetraene-7-one) [93]. In addition, the following known diterpenoids were also isolated: villosin C **(85)**, 12,16-epoxy-11,14-dihydroxy-6-methoxy-17(15→16)-abeo-abieta-5,8,11,13,15-pentanene-3,7-dione **(31)**, uncinatone **(9)**, mandarone E **(70)**, formidiol **(30)**, teuvincenone E **(86)**, teuvincenone F **(10)** and Trichotomone H **(87)** (=12,16-epoxy-17(15→16),18(4→3)-*diabeo*-abieta-3,5,8,12,15-pentaene-7,11,14-trione) [93].

Of the 14 isolated compounds, (10*R*,16*S*)-12,16-epoxy-11,14-dihydroxy-6-methoxy-17(15→16)-abeo-abieta-5,8,11,13-tetraene-3,7-dione **(81)** is a newly discovered naturally occurring compound. All the extraction methods used for the phytochemical analyses of this plant species are shown in Table 11. The cytotoxic activities of these diterpenoids were studied against tumour cell lines BGC-823, Huh-7, KB, KE-97, and Jurkat based on CellTiter Glo™ Luminescent cell viability assay. Of all the tested compounds, trichotomone D **(82)**, F **(84)** and H **(87)**, teuvincenone E and H **(88)**, uncinatone **(9)** and mandarone E **(70)** showed cytotoxic activity. IC_50_ values ranged from 0.83 to 50.99 µM. The most active diterpenoid was found to be Teuvincenone E **(86)**, with IC_50_ values of 3.95, 5.37, 1.18, 1.27, and 0.83 µM against the BGC-823, Huh-7, KB, KE-97, and Jurkat lines, respectively. The authors attribute the high cytotoxic activity of this compound to its rearranged A ring and intact 2-methyl-3-dihydro-furan fragment [93].

In further phytochemical studies, air-dried stems of *Clerodendrum trichotomum* were extracted and chromatographically separated. Eleven compounds were identified, including seven abietane diterpenes: sugiol **(18)**, teuvincenone A **(48)**, teuvincenone B **(89)**, teuvincenone F **(10)**, teuvincenone H **(88)**, uncinatone **(9)** and cyrtophyllone B **(21)** [94]. In further studies on *C. trichotomum* stems, the same authors also identified the diterpenoids villosin B **(90)** and villosin C **(85)**; these demonstrate remarkable cytotoxic activities against tumour cell lines A549, HepG-2, MCF-7 and 4T1 with IC_50_ values ranging from 14.93 to 29.74 µM [95].

Hu et al. (2018) isolated twelve new abietane diterpenoids from *C. trichotomum* roots: 15,16-dehydroteuvincenone G **(91)**, 3-dihydroteuvincenone G **(92)**, 17-hydroxymandarone B **(93)**, trichotomin A **(94)**, 15,16-dihydroformidiol **(95)**, 18-hydroxyteuvincenone E **(96)**, 2α-hydrocaryopincaolide F **(97)**, 15α-hydroxyuncinatone **(98)**, 15α-hydroxyteuvincenone E **(99)**, trichotomin B **(100)**, trichotomside A **(101)** and B **(102)** [96]. As earlier studies indicate that *C. trichotomum* roots possess anti-inflammatory properties [17], all the secondary metabolites isolated by Hu et al. (2018) were tested for their ability to inhibit NO production in LPS-stimulated RAW 264.7 cells, a marker of inflammation [96]. Of the tested substances, 15,16-dehydroteuvincenone G, trichotomin A, 2α-hydrocaryopincaolide F, as well as other isolated compounds, such as villosin C **(85)**, 15-dehydro-17-hydroxycyrtophyllone A **(16)**, demethylcryptojaponol, 6β-hydroxydemethylcryptojaponol and trichotomone **(78)**, exhibited IC_50_ values ranging from 6.0 to 16.1 µM, with 15,16-dehydroteuvincenone G being the most active diterpenoid (IC_50_ value 6.0 µM). It is worth adding that all these active compounds acted at non-cytotoxic concentrations and demonstrated stronger activity than aminoguanidine hydrochloride (IC_50_ 26.2 µM) [96].

**Table 11:** The diterpenoid extraction methods and *C. trichotomum* plant materials for diterpenoid isolation.

Species	Material	Solvent and Extraction Method	Isolated Diterpenoids	References
*C. trichotomum*	Roots	The dried roots were first extracted with petroleum ether/EtOAc (1:1) three times at room temperature, assisted by ultrasonication. After filtration, the filtrate was concentrated at reduced pressure to give a dark brown residue	6-methoxyvillosin C **(79)** 18-hydroxy-6-methoxyvillosin C **(80)** (10*R*,16*S*)-12,16-epoxy-11,14-dihydroxy-6-methoxy-17(15→16)-abeo-abieta-5,8,11,13-tetraene-3,7-dione **(81)** Trichotomone D **(82)** (10*R*,16*R*)-12,16-epoxy-11,14,17-trihydroxy-17(15→16),18(4→3)-diabeo-abieta-3,5,8,11,13-pentaene-2,7-dione **(83)** Trichotomone F **(84)** 12,16-epoxy-11,14-dihydroxy-6-methoxy-17(15→16)-abeo-abieta-5,8,11,13,15-pentanene-3,7-dione **(31)** Uncinatone **(9)** Mandarone E **(70)** Formidiol **(30)** Teuvincenone E **(86)** Teuvincenone F **(10)** Trichotomone H **(87)** Villosin C **(85)**	[93]
*C. trichotomum*	Roots	Dried roots of *C. trichotomum* were exhaustively extracted with petroleum ether/EtOAc (1:1) at room temperature, yielding a semi-dry residua	Trichotomone **(78)**	[92]
*C. trichotomum*	Stems	The air-dried stems were cut into small pieces and extracted with 85% EtOH under reflux three times. The combined extracts were filtered and the solvent was removed under reduced pressure with a rotary evaporator at 60 °C to obtain a brown crude extract.	Sugiol **(18)** Teuvincenone F **(10)** Teuvincenone A **(48)** Teuvincenone H **(88)** Uncinatone **(9)** Teuvincenone B **(89)** Cyrtophyllone B **(21)**	[88]
*C. trichotomum*	Stems	The air-dried stems were cut into small pieces and extracted with 85% EtOH under reflux three times. The combined extracts were filtered and the solvent was removed under vacum to obtain a crude extract.	Villosin B **(90)** Villosin C **(85)** Cyrtophyllone B **(21)** Uncinatone **(9)** Teuvincenone B **(89)** Sugiol **(18)** Teuvincenone F **(10)** Teuvincenone A **(48)** Teuvincenone H **(88)**	[95]
*C. trichotomum*	Roots	Cut and air-dried roots were extracted under reflux with 95% EtOH Extract was filtered and organic solvents evaporated to receive the crude residue	15,16-dehydroteuvincenone G **(91)** 3-dihydroteuvincenone G **(92)** 17-hydroxymandarone B **(93)** Trichotomin A **(94)** 15,16-dihydroformidiol **(95)** 18-hydroxyteuvincenone E **(96)** 2α-hydrocaryopincaolide F **(97)** 15α-hydroxyuncinatone **(98)** 15α-hydroxyteuvincenone E **(99)** Trichotomin B **(100)** Trichotomside A **(101)** Trichotomside B **(102)**	[96]

## 3. The Latest Data

Woody branches and healthy stems of *Clerodendrum bracteatum* were the plant materials used for the extraction, isolation and purification to identify two new abietane diterpenes compounds, which are defined as: (10*S*,16*S*)-12,16-epoxy-17(15→16)-*abeo*-3,5,8,12-abietatetraen-7,11,14-trione (**103**) and 11,14,16-trihydroxy-6,12-dimethoxy-17(15→16)-abeo-5,8,11,13- abietatetraen-3,7-dione (**104**) [97]. The extraction method used for the phytochemical analyses of this plant species is shown in Table 12. Both phytochemicals have an *abeo*-abietane structure, the first of which has *p*-quinone and *p*-benzoquinone moieties. In compound **104,** four methyls, two ketones at C-3 and C-7, as well as the presence of a methine group at C-16 were detected. According to Li et al. (2021), two newly isolated structures (**103** and **104**) have the strongest antioxidant and cytotoxic activities against HL-60 and A-549 tumour cell lines among seven isolated diterpenes [97].

Interesting new data on diterpenes were published in the work of Qi et al. in 2021 [98]. The authors successfully undertook the extraction of *Clerodendrum chinense* roots, which resulted in the isolation and identification of 6 new diterpenes: Clerodenoids A–F (**105–110**) (Table 13). All of these compounds have an aromatised C ring. It is worth noting that structures **106–108** are the rearranged abietane diterpenoids sharing a 17(15→16)-abeo-abietane skeleton, while compounds **109–110** are 17(15→16),18(4→3)-diabeo-abietane moieties. Furthermore, compound **110** has a persubstituted Δ3 double bond and methylhydroxyl function in the A ring. All six newly isolated diterpenes were examined towards antiproliferative activities against HL-60 and A-549 human tumour cell lines [98]. The most active diterpene was compound **110** demonstrating IC_50_ values at 1.36 and 1.00 µM against HL-60 and A-549 cell lines, respectively [98].

*Clerodendrum infortunatum* aerial parts were used for extraction and isolation of terpenoid compounds [76]. Among various known compounds, two previously unknown diterpenes were isolated and identified as (5*R*,10*S*,16*R*)-11,16,19-trihydroxy-

12-O-β-D-glucopyranosyl-(1→2)-β-D-glucopyranosyl-17(15→16),18(4→3)-*diabeo*-3,8,11,13-abietatetraene-7-one (**111**) and (5*R*,10*S*,16*R*)-11,16-dihydroxy-12-O-β-D-glucopyranosyl-(1→2)-β-D-glucopyranosyl-17(15→16),18(4→3)-*diabeo*-4-carboxy-3,8,11,13-abietatetraene-7-one (**112**) [76]. The extraction method used for the phytochemicals analyses of this plant species is shown in Table 6. Inhibition of converting carbohydrates into monosaccharides is considered to be an adjunct to the treatment of type 2 diabetes. Therefore, it was justified by the authors to investigate this activity among isolated compounds. The isolated secondary metabolites were tested for their ability to inhibit α-amylase and α-glucosidase. Compound **111** ((5*R*,10*S*,16*R*)-11,16,19-trihydroxy-12-O-β-D-glucopyranosyl-(1→2)-β-D-glucopyranosyl-17(15→16),18(4→3)-*diabeo*-3,8,11,13-abietatetraene-7-one) inhibited these enzymes activity with an IC_50_ value of 18.5 and 24.6 µM, respectively. (5R,10S,16R)-11,16-dihydroxy-12-O-β-D-glucopyranosyl-(1→2)-β-D-glucopyranosyl-17(15→16),18(4→3)-*diabeo*-4-carboxy-3,8,11,13-abietatetraene-7-one demonstrated weaker activity (IC_50_ = 64.6 and 78.3 µM) [76]. Additionally, the studied compounds were tested for acethyl- and buthyrylcholinesterase (AChE and BChE) inhibition, showing weak activity against AChE with an IC_50_ of 191 and 139 µM, respectively [76].

The diterpenes present in *C. inerme* [69] were isolated from the dried roots of *Clerodendrum bungei* crolerodendrum A (**43**) and B (**42**) for the first time in this species [20,22].

Compound **42** is known for its antioxidant properties [70] and exhibits significant inhibition against the α-glucosidase enzyme with an IC_50_ value of 17 µM [39].

The biological activities of the diterpenoids isolated from the *Clerodendrum* genus are summarised in Table 14.

## 4. Concluding Remarks

The members of the genus *Clerodendrum*, of the family *Lamiaceae*, are rich in diterpenoid secondary metabolites, both in the aerial parts and the roots. Due to their moderate, and in some cases strong, biological activities, these diterpenoids are interesting experimental objects. This is particularly true for in vivo pharmacological evaluation. Some of the diterpenes isolated from *Clerodendrum* spp. are structurally similar to the more highly active phytocompounds; however, they have not been tested for their potential biological activities. This is an important area for further study, as both infectious and civilization diseases, such as cancer, require the search for new therapeutically active structures. The new metabolites obtained from *Clerodendrum* spp. demonstrate high pharmacological potential, and could be an interesting object of further studies, particularly plant in vitro culture aimed at optimizing the cultivation conditions to increase biomass and secondary metabolite production, especially diterpenes. These biotechnological investigations should determine the effect of culture type (callus, shoot, modified root) and growth conditions such as basal medium and light wavelength, regardless of climatic conditions, season and environmental pollution.

Another interesting area of research concerning the diterpenes from *Clerodendrum* could be the chemical modification of the isolated phytocompounds. These would include the production of semisynthetic analogues with enhanced biological activities, and improved bioavailability or safety [99,100].

Another equally interesting area of research into these diterpenes is biotransformation [101]. Biotransformation is a very useful tool for the structural modification of natural products with complex chemical structures. Research into the biotransformation of metabolic pathways is essential to understand the potential toxicity and efficacy of new drug candidates and should be a mandatory part of preclinical studies [102].

## Figures and Tables

**Table 1:** The diterpenoid extraction methods and *C. bungei* plant material for diterpenoid isolation.

Species	Material	Solvent and Extraction Method	Isolated Diterpenoids	References
*C. bungei*	Roots	70% aqueous acetone. Dried roots extracted in room temperature. Solvent removed in vacuo.	12-O-D-glucopyranosyl-3,11,16-trihydroxyabieta-8,11,13-triene **(3)** 3,12-O-D-diglucopyranosyl-11,16-dihydroxyabieta-8,11,13-triene **(4)** 19-O-D-carboxyglucopyranosyl-12-O-D-glucopyranosyl-11,16-dihydroxyabieta-8,11,13-triene **(5)** 11,16-dihydroxy-12-O-D-glucopyranosyl-17(15f16),18(4f3)-abeo-4-carboxy-3,8,11,13-abietatetraen-7-one **(6)** 19-hydroxyteuvincenone F **(7)** ajugaside A **(8)** uncinatone **(9)**	[36]
*C. bungei*	Stem	**EtOH**. Dried and pulverised stems were extracted three times with hot solvent. The solvent was removed in vacuo.	bungone A **(1)** bungone B **(2)** sugiol **(18)** uncinatone **(9)** teuvincenone F **(10)**	[35]
*C. bungei*	Roots	**95% EtOH.** Air-dried powdered root parts were extracted three times at room temperature. The solvent was removed in vacuo. The crude EtOH extract was suspended in hot water and then partitioned with EtOAc four times.	3β-(β-D-glucopyranosyl)isopimara-7,15-diene-11α,12a-diol **(11)** 16-O-β-D-glucopyranosyl-3β-20-epoxy-3-hydroxyabieta-8,11,13-triene **(12)**	[34]
*C. bungei*	Roots	**70% aqueous acetone.** Air-dried roots were extracted at room temperature. The extract was filtered through a Buchner funnel using Whatman No. 1 filter paper. The solvent was removed in vacuo.	12-O-β-D-glucopyranosyl-3,11,16-trihydroxyabieta-8,11,13-triene **(3)** 3,12-O-β-D-diglucopyranosyl-11,16-dihydroxyabieta-8,11,13-triene **(4)** ajugaside A **(8)** uncinatone **(9)** 19-hydroxyteuvincenone F **(7)**	[37]
*C. bungei*	Roots	**EtOH.** Dried roots were extracted three times under conditions of reflux (every 2 h). Organic extracts were combined. The solvent was removed under reduced pressure. **EtOH.** Dried roots were extracted three times at room temperature for three times. The residue was re-suspended in L water and partitioned successively with EtOAc and *n*-BuOH.	bungnate A **(13)** bungnate B **(14)** 15-dehydrocyrtophyllone A **(15)** 15-dehydro-17-hydroxycyrtophyllone A **(16)** 12,16-epoxy-11,14,17-trihydroxy-6-methoxy-17(15→16)-abeo-abieta-5,8,11,13-tetraene-7-one **(16)** cyrtophyllone A **(17)** villosin C **(85)** teuvincenone F **(10)** 19-hydroxyteuvincenone F **(7)** mandarone E **(70)** 12-O-β-D-glucopyranosyl-3,11,16-trihydroxyabieta-8,11,13-triene **(3)** uncinatone **(9)** crolerodendrum B **(42)** crolerodendrum A **(43)**	[38] [39]

**Table 2:** The diterpenoid extraction method and *C. cyrtophyllum* plant material for diterpenoid isolation.

Species	Material	Solvent and Extraction Method	Isolated Diterpenoids	References
*C. cyrtophyllum*	Stem	EtOH. The whole plant was dried and pulverised. It was extracted three times with hot solvent. The solvent was recovered in vacuo.	Cyrtophyllone A **(17)** Cyrtophyllone B **(21)** Teuvincenone F **(10)** Uncinatone **(9)**	[42]
			Sugiol **(18)**	

**Table 3:** The diterpenoid extraction method and *C. eriophyllum* plant material for diterpenoid isolation.

Species	Material	Solvent and Extraction Method	Isolated Diterpenoids	References
*C. eriophyllum*	Roots	1:1 MeOH/CH_2_Cl_2_; MeOH. Roots were dried and pulverised. They were extracted by cold percolation at room temperature using three portions of 1:1 MeOH/CH_2_Cl_2_ and then extracted with 100% MeOH once.	12-hydroxy-8,12-abietadiene-3,11,14-trione **(22)** Royleanone **(23)** Taxodione **(24)** 6-deoxy-taxodione **(25)** Sugiol **(18)** Ferruginol **(26)** 6-hydroxysalvinolone **(27)** 6,11,12,16-tetrahydroxy-5,8,11,13-abietatetra-en-7-one **(28)** Uncinatone **(9)** 11-hydroxy-8,11,13-abietatriene-12-O-β-xylopyranoside **(29)**	[47]

**Table 4:** The diterpenoid extraction method and *C. formicarum* plant material for diterpenoid isolation.

Species	Material	Solvent and Extraction Method	Isolated Diterpenoids	References
*C. formicarum*	Leaves	EtOH. Leaves were dried under shade for a week. Powdered material was soaked in ethanol for six days. The resulting extract was concentrated by evaporation under vacuum distillation.	formidiol **(30)** 12,16-epoxy-11,14-dihydroxy-6-methoxy-17(15→16)-abeo-abieta-5,8,11,13,15-pentanene-3,7-dione **(31)**	[65]

**Table 5:** The diterpenoid extraction methods and *C. inerme* plant materials for diterpenoid isolation.

Species	Material	Solvent and Extraction Method	Isolated Diterpenoids	References
*C. inerme*	Leaves	Hexane–EtOAc. Dried and finely powdered aerial parts of the plant were extracted with hexane—EtOAc (40:60)—fraction 1; and hexane—EtOAc (25:75)—fraction 2.	Inermes A **(39)** Inermes B **(40)** 14,15-dihydro-15β-methoxy-3-epicaryoptin **(41)**	[69]
	Aerial parts	MeOH. Dried and powdered aerial parts were extracted in a Soxhlet apparatus for 15 h. The extract was concentrated under red. pres., dil. with H_2_O and the liberated solid was exhaustively extracted with Et_2_O.	Cleroinermin **(32)**	[66]
	Leaves	MeOH. Air-dried and powdered roots were extracted three times by sonication for 30 min., concentrated under reduced pressure. They were suspended in water and successively partitioned with dichloromethane three times.	Crolerodendrum B **(42)** Crolerodendrum A **(43)** Uncinatone **(9)** Harwickiic acid **(44)** 14,15-dihydro-15β-methoxy-3-epicaryoptin **(41)**	[70]
	Aerial parts	The shade-dried, crushed aerial parts were percolated with *n*-hexane (three times) for 24 h. The resulting extract was then concentrated under vacuum to obtain a residue	clerodendrin B **(33)** 3-epicaryoptin **(34)** Clerodendrin C **(35)** 2-acetoxyclerodendrin B **(36)** 15-hydroxyepicaryoptin **(37)**	[68]

**Table 6:** The diterpenoid extraction methods and *C. infortunatum* plant materials for diterpenoid isolation.

Species	Material	Solvent and Extraction Method	Isolated Diterpenoids	References
*C. infortunatum*	Aerial parts Leaves	The shade was dried, the crushed aerial parts were exhaustively extracted with *n*-hexane (3×). The extract was then	Clerodin **(45)** (5*R*,10*S*,16*R*)-11,16,19-trihydroxy- 12-O-β-D-glucopyranosyl- (1→2)-β-D-glucopyranosyl- 17(15→16),18(4→3)-*diabeo*- 3,8,11,13-abietatetraene-7-one (5*R*,10*S*,16*R*)-11,16-dihydroxy- 12-O-β-D-glucopyranosyl-(1→2)- β-D-glucopyranosyl-17(15→16),18(4→3)- *diabeo*-4-carboxy-3,8,11,13-abietatetraene-7-one	[68] [76]
		concentrated in vacuo to ∼250 mL The leaves were extracted with **acetone**. The extract was concentrated in vacuo. The residue was solvated in a solution of water: methanol and partitioned with ethyl acetate and *n*-butanol, respectively.		
	Leaves	*n*-hexane/MeOH. The leaves were dried in the shade at room temperature and then ground in an electric grinder. The leaf powder was soaked in *n*-hexane for 72 h, shaken occasionally. The extract was filtered, concentrated in a rotary vacuum evaporator, then partitioned into hexane and methanol. After filtration, the leaf powder residue was further extracted with methanol and concentrated. This extract was further partitioned with hexane, ethyl acetate, and butanol.	clerodin **(45)** 15-methoxy-14,5- dihydroclerodin **(46)** 15-hydroxy-14,15-dihyroclerodin **(47)**	[75]

**Table 7:** The diterpenoid extraction method and *C. kaichianum* plant material for diterpenoid isolation.

Species	Material	Solvent and Extraction Method	Isolated Diterpenoids	References
*C. kaichianum*	Stem	**EtOH.** The air-dried and powdered stems was extracted with 75% aq. EtOH three times each at 75 °C for 4 h. The EtOH extracts were combined and evaporated.	17-hydroxyteuvincenone G **(51)** 17-hydroxyteuvincen-5(6)-enone G **(52)** Teuvincenone A **(48)** 11,14-dihydroxyabieta-8,11,13-trien-7-one **(49)** Dehydroabietan-7-one **(50)** Sugiol **(18)**	[80]
			(16R)-12,16-epoxy-11,14,17-trihydroxy-17(15→16)-abeo-8,11,13-abietatrien-7-one **(58)** Villosin A **(53)** Salvinolone **(54)** 14-deoxycoleon U **(55)** 5,6-dehydrosugiol **(56)** Coleon U **(57)**	[81]

**Table 8:** The diterpenoid extraction method and *C. kiangsiense* plant material for diterpenoid isolation.

Species	Material	Solvent and Extraction Method	Isolated Diterpenoids	References
*C. kiangsiense*	Stem	EtOH. The air-dried and powdered stems extracted by 90% ethanol three times at 65 °C. The solvents were combined and evaporated to dryness under vacuum.	12-methoxy-6,11,14,16-tetrahydroxy-17(15→16)-abeo-5,8,11,13-abietatetraen-3,7-dione **(59)** Mandarone A **(60)** Taxusabietane A **(61)** 12-O-demethyl-cryptojaponol **(62)** Cryptojaponol **(63)** 11,14-dihydroxy-8,11,13-abietatrien-7-one **(64)** Fortunin E **(65)** Fortunin F **(66)**	[82]

**Table 9:** The diterpenoid extraction method and *C. mandarinorum* plant material for diterpenoid isolation.

Species	Material	Solvent and Extraction Method	Isolated Diterpenoids	References
*C. mandarinorum*	Stem	EtOH. The naturally dried and pulverised stems were extracted with hot EtOH three times. The solvent was removed in vacuo.	Mandarone D **(69)** Mandarone E **(70)** Mandarone F **(71)** Mandarone G **(72)** Mandarone H **(73)**	[85]
			Mandarone A **(60)** Mandarone B **(67)** Mandarone C **(68)**	[83]

**Table 10:** The diterpenoid extraction method and *C. splendens* plant material for diterpenoid isolation.

Species	Material	Solvent and Extraction Method	Isolated Diterpenoids	References
*C. splendens*	Aerial parts	Dried powdered aerial parts of C. splendens were successively and separately extracted for 48 h with *n*-hexane, CHCl_3_, CHCl_3_–MeOH (9:1), and MeOH, by exhaustive maceration	2α-acetoxy-3β-(2′,3′-diacetoxy-2′-methyl)-butanoyloxy-14-hydro-15-hydroxyclerodin **(74)** 3β,15-dihydroxy-14-hydro-clerodin **(75)** 2α,15-dihydroxy-3β-(2′-hydroxy-2′-methyl-3′-acetoxy)-butanoyloxy-6α,18-diacetoxy-4α,17-epoxy-clerodan-11,16-lactone **(76)** 3β,14S,15-trihydroxy-6α,18-diacetoxy-4α,17-epoxy-clerodan-11,16-lactone **(77)**	[91]

**Table 12:** The diterpenoid extraction methods and *Clerodendrum bracteatum* plant materials for diterpenoid isolation.

Species	Material	Solvent and Extraction Method	Isolated Diterpenoids	References
*C. bracteatum*	Stems	EtOH. Cut and air-dried stems were extracted under reflux with 90% Ethanol and evaporated to to afford a gummy residue. The crude extract was suspended in water and fractionated with EtOAc and *n*-BuOH.	(10*S*,16*S*)-12,16-epoxy-17(15→16)-*abeo*-3,5,8,12-abietatetraen-7,11,14-trione (**103**) 11,14,16-trihydroxy-6,12-dimethoxy-17(15→16)-abeo-5,8,11,13- abietatetraen- 3,7-dione (**104**)	[97]

**Table 13:** The diterpenoid extraction methods and *Clerodendrum chinense* plant materials for diterpenoid isolation.

Species	Material	Solvent and Extraction Method	Isolated Diterpenoids	References
*C. chinense*	Roots	EtOH. The dried roots were soaked with 95% EtOH (20 L × 3) and the residue was suspended in water followed by extraction with EtOAc to afford an EtOAc extract	Clerodenoid A Clerodenoid B Clerodenoid C Clerodenoid D Clerodenoid E Clerodenoid F	[98]

**Table 14:** The biological activities of diterpenoids isolated from *Clerodendrum* genus.

Compound Name	Occurence in *Clerodendrum* Species	Biological Activity	References of the Active Compounds
uncinatone	*C. bungei* (roots), *C.* *eriophyllum* (roots), *C. inerme* (aerial parts) *C. trichotomum* (roots)	Cytotoxic; inhibition the cell proliferation and induction cell-cycle G2/M phase arrest; anti-complement activity on the classical pathway complement system, antileishmanial, antioxidant;	[36,41,70]
12-O-β-D-glucopyranosyl-3,11,16-trihydroxyabieta-8,11,13-triene; 3,12-O-β-D-diglucopyranosyl-11,16-dihydroxyabieta- 8,11,13-triene; 19-hydroxyteuvincenone F	*C. bungei* (roots)	anti-complement activity on the classical pathway complement system;	[37]
16-O-β-D-D-glucopyranosyl-3β-20-epoxy-3-hydroxyabieta-8,11,13-triene	*C. bungei* (roots)	Cytotoxic	[34]
15-Dehydrocyrtophyllone A	*C. bungei* (roots)	ACE (angiotensin-converting enzyme) inhibition;	[38]
cyrtophyllone B	*C. cyrtophyllum* (whole plant)	cytotoxic; acethyl- and butyrylcholineesterase inhibition;	[43]
royleanone; taxodione; 6-deoxy-taxodione; sugiol; ferruginol; 6-hydroxysalvinolone; 6,11,12,16-tetrahydroxy-5,8,11,13-abietatetra-en-7-one;	*C.eriophyllum* (roots)	Cytotoxic, weak antibacterial; cytotoxic, antibacterial, acethyl- and butyrylcholineesterase inhibition; antibacterial and antileishmanial antioxidant, antibacterial, antiviral, anticancer, anti-tumor and anti-inflammatory, antiviral, cytotoxic; antibacterial, antifungal, antimalarial, cytotoxic; apoptose induction, antifungal, cytotoxic; antibacterial, antifungal, cytostatic, cytotoxic;	[50,51] [30,50,51,52] [53] [55,56,57,58] [59,60,61,62] [47,63] [64]
12,16-epoxy-11,14-dihydroxy-6-methoxy-17(15→16)-abeo-abieta-5,8,11,13,15-pentanene-3,7-dione	*C. formicarum* (leaves)	antiproliferative	[43]
cleroinermin; clerodendrin B; 3-epicaryoptin; clerodendrin C; 2-acetoxyclerodendrin B; 15-hydroxyepicaryoptin; clerodermic acid harwickiic acid crolerodendrum B	*Clerodendrum inerme* (aerial parts) *Clerodendrum inerme* and *C. bungei* (aerial parts)	Neuroprotective, insecticidal, antiproliferative and apoptose induction antileishmanial, cytotoxic, antioxidant, antioxidant, α-glucosidase inhibition	[67] [68] [72] [74] [70] [39]
clerodin; 15-methoxy-14,15-dihydroclerodin; 15-hydroxy-14,15-dihyroclerodin	*C. infortunatum* (leaves)	insecticidal	[68,75,77]
(5*R*,10*S*,16*R*)-11,16,19-trihydroxy- 12-O-β-D-glucopyranosyl-(1→2)-β-D-glucopyranosyl-17(15→16),18(4→3)-*diabeo*-3,8,11,13-abietatetraene-7-one (5R,10S,16R)-11,16-dihydroxy-12-O-β-D-glucopyranosyl-(1→2)-β-D-glucopyranosyl-17(15→16),18(4→3)-*diabeo*-4-carboxy-3,8,11,13-abietatetraene-7-one 17-hydroxyteuvincenone G; 17-hydroxyteuvincen-5(6)-enone G; villosin A; salvinolone; 14-deoxyloleon U; 5,6-dehydrosugiol; coleon U; (16R)-12,16-epoxy-11,14,17-trihydroxy-17(15→16)-abeo-8,11,13-abietatrien-7-one	*C. infortunatum* (leaves) *C. kaichianum* (steams)	Acethyl- and buthyrylcholinesterase (AChE and BChE) inhibition, α-amylase and α-glucosidase inhibition cytotoxic	[76] [80,81]
taxusabietane A; cryptojaponol; 11,14-dihydroxy-8,11,13-abietatrien-7-one	*C. kiangsiense* (aerial parts)	Anti-inflammatory Cytotoxic Cytotoxic, antiprotozoal	[86] [87] [88,89]
2α-acetoxy-3β-(2′,3′-diacetoxy-2′-methyl)-butanoyloxy-14-hydro-15-hydroxyclerodin; 2α,15-dihydroxy-3β-(2′-hydroxy-2′-methyl-3′-acetoxy)-butanoyloxy-6α,18-diacetoxy-4α,17-epoxy-clerodan-11,16-lactone	*C. splendens*	Antiproliferative	[91]
trichotomone D, F and H; teuvincenone E and H, uncinatone; mandarone E villosin B and C 15,16-dehydroteuvincenone G; trichotomin A; 2α-hydrocaryopincaolide F; villosin C; 15-dehydro-17-hydroxycyrtophyllone A; demethylcryptojaponol; 6β-hydroxydemethylcryptojaponol; trichotomone (10*S*,16*S*)-12,16-epoxy-17(15→16)-*abeo*-3,5,8,12-abietatetraen-7,11,14-trione 11,14,16-trihydroxy-6,12-dimethoxy-17(15→16)-abeo-5,8,11,13-abietatetraen- 3,7-dione Clerodenoid A Clerodenoid D Clerodenoid E Clerodenoid F	*C. trichotomum* (roots) (steams) (roots) *Clerodendrum bracteatum* (stems) *Clerodendrum chinense* (roots)	Cytotoxic Cytotoxic Anti-inflammatory Antioxidant, cytotoxic Cytotoxic	[92,93] [95] [96] [97] [98]

## Data Availability

Not applicable.
